# Dissemination of trial results to study participants in Cambodia, Ethiopia and Pakistan: a qualitative study

**DOI:** 10.1136/bmjopen-2026-119698

**Published:** 2026-08-10

**Authors:** Sarah A Cassidy-Seyoum, Bipin Adhikari, Muthoni Mwaura, Asia Khan, Shan-E- Zehra Zaidi, Zahida Azizullah, Bushra Qurashi, Kansite Gellebo, Samuel Alemu Bamboro, Mom Ean, Rupam Tripura, Hem Vattanak, Thy Vanda, Kem Sovann, Ou Simvieng, Phal Chanpheakdey, Hellen Mnjala, Grant Lee, Najia Ghanchi, Phaik Yeong Cheah, Ric N Price, M. Asim Beg, Tamiru Shibiru Degaga, Lek Dysoley, Kamala Thriemer

**Affiliations:** 1 Global and Tropical Health Division, Menzies School of Health Research, Charles Darwin University, Darwin, Northern Territory, Australia; 2 Mahidol Oxford Tropical Medicine Research Unit, Faculty of Tropical Medicine, Mahidol University, Bangkok, Thailand; 3 Centre for Tropical Medicine and Global Health, Nuffield Department of Clinical Medicine, University of Oxford, Oxford, England, UK; 4 Department of Health Ethics and Society, Care and Public Health Research Institute (CAPHRI), Maastricht University, Maastricht, Netherlands; 5 Department of Pathology and Laboratory Medicine, The Aga Khan University, Karachi, Sindh, Pakistan; 6 Department of Sociology and Social Anthropology, Arba Minch University, Arba Minch, Ethiopia; 7 College of Medicine & Health Sciences, Arba Minch University, Arba Minch, Ethiopia; 8 National Center for Parasitology Entomology and Malaria Control, Phnom Penh, Phnom Penh, Cambodia; 9 School of Public Health, National Institute of Public Health, Phnom Penh, Cambodia

**Keywords:** QUALITATIVE RESEARCH, Community Participation, MEDICAL ETHICS, Clinical Trial

## Abstract

**Abstract:**

**Objectives:**

Dissemination of study results to research participants is an ethical imperative, yet it is rarely implemented in resource-limited settings partly due to a lack of clear guidance. The purpose of this study was to evaluate and explore dissemination activities that were conducted as part of a multi-site clinical trial of *Plasmodium vivax* treatment options, informing future dissemination strategies in similar resource-limited settings.

**Design:**

Qualitative study using focus group discussions (FGDs) and an inductive–deductive thematic analysis approach.

**Setting:**

Clinical trial study site catchment areas in Cambodia, Ethiopia and Pakistan.

**Participants:**

Dissemination attendees were invited to participate in FGDs, a subset agreeing and able to participate. There were 25 participants in Cambodia, 34 in Ethiopia and 23 in Pakistan.

**Results:**

The dissemination activities were well accepted by participants despite some expectations in receiving individual results rather than only aggregated trial results and suggestions for broader community dissemination. Dissemination participants perceived having learnt from the dissemination, demonstrating their knowledge of the trial and its results and attributing it to the dissemination. Dissemination activities may have failed to correct certain misconceptions, including the blurred distinction between clinical care and trial-related care; however, the dissemination had positive impacts, including making participants feel part of a larger endeavour.

**Conclusion:**

Contributing to the limited data on dissemination in resource-limited settings, our findings provide context-informed recommendations that can be applied to similar settings. Our recommendations underscore the importance of tailoring activities according to participant preferences, such as potentially providing individual results and implementing broader community dissemination.

**Trial registration number:**

NCT04411836.

**STRENGTHS AND LIMITATIONS OF THIS STUDY**

- A key strength of this study is that it includes three different settings and contexts, providing richer data and opportunities for triangulation; however, different dissemination evaluation methods were used in the different settings, making cross-country comparisons difficult.
- This study draws on previous literature, available acceptability frameworks and the data generated in this study to develop a framework aimed at guiding future evaluation of dissemination. The development of the framework was both informed by and contributed to our data analysis.
- The subset of attendees who participated in the study are reflective of the broader trial population.
- This study evaluated the dissemination experience from a participant standpoint, excluding the perspectives of family members, broader community, country partners and site study teams.

## Introduction

 Clinical research aims to improve health outcomes by assessing and comparing health interventions, including novel treatments and diagnostic tools. Research participants are central to this endeavour, and there is a duty to ensure that they are protected and should potentially benefit from the interventions being studied.^1–3^ The implementation of clinical research and trials is governed by global ethics standards,^1 2^ which extend beyond ensuring scientific validity and minimising harm to emphasise respect for persons, including informed consent, privacy and fair distribution of risks and benefits. There is increasing recognition that global ethics standards should also extend beyond recruitment and data collection to cover how research relationships are conducted and concluded.^1 4 5^

Since 2013, the World Medical Association’s Helsinki Declaration states that medical research results should be published and disseminated and specifically that research participants should have the option of being informed of study results.^1^ This mandate is now incorporated into regulations in Australia, Canada, the European Union and the United States, highlighting the growing importance of participant dissemination as part of health research standards.^6–9^

Dissemination of study results to participants as part of community engagement is increasingly recognised as an ethical and moral imperative in clinical research^1 10–12^ and included in ethics guidelines^13^; however, in practice, it is rarely implemented globally,^10 11 14–16^ including in the context of high-income countries.^17 18^ This is illustrated by most literature focusing largely on pre-trial and during-trial community engagement.^19 20^ In a 2016 survey, only 27% of clinical trial investigators reported having conducted participant dissemination, and 33% had no intention of sharing results with participants.^16^ Reasons for the limited uptake of participant dissemination include a lack of prioritisation by researchers, often based on an assumption that patients are not interested or that they could not understand, limited institutional support and funding and other operational and cultural barriers.^10 11 16^ The latter includes difficulties reaching patients, long periods between trial completion and the generation of the results and a lack of early planning and knowledge of best practices.^10 11 16^

Literature reviews indicate that participants want to be informed of study results, regardless of the outcome of the study.^10 14^ When participant dissemination does occur, the most used method has been the posting of lay summaries in line with participant preferences for written materials.^10^ In low-income and middle-income countries (LMICs), in-person group presentations or meetings are the main method for dissemination^10^; however, there is a scarcity of evidence, especially in LMICs, as well as a lack of agreed best practices.

In the global health and infectious disease landscape, medical research is increasingly conducted in LMICs,^21 22^ where the disease burden is high. Community engagement, including post-trial participant dissemination, is especially important to build trust because clinical research is often led by international research organisations or institutions.^3 19 23^ Historically, and in some cases today, these external organisations have come into LMICs, conducted their trial and left without informing local stakeholders and participants of study findings.^11 24^ This practice is ethically questionable and detrimental to researchers’ relationships with communities, impacting future clinical research and trust in science.^24–26^ Today, most researchers are aware of the responsibility for participant dissemination; however, a lack of knowledge of preferences, best practices and guidance is often a barrier to implementation.^11^

As part of a multi-site clinical trial on the effectiveness of treatment options for patients with *Plasmodium vivax* malaria,^27^ trial participants were surveyed at enrolment regarding their desire to be informed about study results and their preferences for dissemination.^28^ Based on these preferences, context-specific dissemination activities were developed and conducted in three of the four study site countries where participants showed interest in receiving the results: Cambodia, Ethiopia and Pakistan.

The objective of this qualitative study was to evaluate and explore the perceived value of dissemination activities while informing future dissemination strategies in similar resource-limited settings.

## Methods

### Study context

This evaluation study was conducted for the dissemination of results from a clinical trial (the EFFORT study, NCT04411836), which assessed novel treatment options for *P. vivax* malaria, including high-dose primaquine (7 mg/kg total dose) and single-dose tafenoquine (300 mg fixed dose) compared with the low-dose primaquine treatment (3.5 mg/kg total dose). A total of 960 patients were recruited at seven study sites in four countries: three in Cambodia, one in Ethiopia, one in Indonesia and two in Pakistan. All patients included in the trial had uncomplicated *P. vivax* malaria, normal glucose-6-phosphate dehydrogenase (G6PD) levels and attended regular follow-up visits, which included a medical examination and blood collection. Weekly visits were conducted until day 42, and then patients were reviewed monthly until completing a 6-month follow-up. The trial recruited participants between April 2021 and March 2025. The full trial results have been published elsewhere,^27^ as have our results from dialogues with policymakers.^29–31^ Study site characteristics are detailed in table 1.

**Table 1:** EFFORT dissemination clinical trial study site characteristics

Characteristic	Cambodia	Ethiopia	Pakistan
*P. vivax* malaria burden	Path to elimination 1307 cases in 2023 Transmission focused in the forest and forest fringe^55 56^	Burden reduction 904 398 cases in 2023 Unstable and increasing transmission (low and highlands)^55 57^	Burden reduction 1 761 637 cases in 2023 High in border areas and increasing transmission due to flooding^55 58^
Clinical trial site location	Phnom Kravanh District Hospital, Phnom Kravanh, Pursat, Western Cambodia (45.5% of Cambodia trial participants)* Siem Pang Health Centre, Siem Pang, Steung Traeng, Northeastern Cambodia (53.2% of Cambodia trial participants)* Chambak Health Centre, Phnom Srouch, Kampong Speu, Southwestern Cambodia (1.3% of Cambodia trial participants)	Arba Minch General Hospital Malaria Research and Treatment Centre, Arba Minch town, Arba Minch, Gamo Zone, Southern Ethiopia Region*	Health facility near Thatta Civil Hospital, Sindh Province, Pakistan (90.8% of Pakistan trial participants)* Khidmat-e-Alam Medical Centre, Nazimabad, Karachi, Pakistan (9.2% of Pakistan trial participants)
Study site catchment area	Phnom Kravanh: 50 000* Siem Pang: 25 000* Chambak: 4000	4million^59^	Thatta: 980 000* Karachi: 3 million
Study site topography	Phnom Kravanh: semi-urban surrounded by forest fringe* Siem Pang: forest fringe surrounded by forest reserves and a national park* Chambak: forest and forest fringe	Urban and semi-urban area with a comprehensive specialised hospital, three primary care hospitals, two health centres, over 30 private facilities, including a private hospital^59^	Thatta: rural, agricultural area* Karachi: urban area
Patient type	Forest-goers, mobile migrant populations, farmers and soldiers	Patients with diverse socio-economic backgrounds and varied health literacy levels	Thatta: agriculturalists and farmers Karachi: heterogeneous patient type*
Malaria care seeking	Travelling relatively far to *public* health centres or hospitals known to have suboptimal quality of care but free malaria care	Varied travel distances to *public* (for free care) or *private* healthcare facilities with supply and stockout challenges	Thatta: travelling long distances to health facilities, mostly seeking care from *private* facilities and making use of traditional remedies^60^*
Radical cure policy	Access to radical cure (low-dose 7-day primaquine) at health centres with routine administration of primaquine after G6PD testing^61 62^	Radical cure with primaquine (low-dose 14-day) recommended without G6PD testing, but primaquine is not always available^63^	Radical cure with primaquine (low-dose 14-day) recommended; only available in public facilities and inconsistent administration^64 65^
Trial enrolment period	April 2021–February 2023	January 2022–December 2023	April 2023–September 2024
Result dissemination date	Phnom Kravanh: 17-28 February 2025* Siem Pang: 27–31 March 2025*	3 July 2025	23 July 2025
Participant preferences for dissemination^28^	33.3% of participants interested in receiving aggregate study results 37.9% preferred a phone call to explain the results 77.6% of patients preferred a summary in their language. Reason for dissemination: 41.4% identified to understand the benefit of the study to the community	100.0% of participants interested in receiving aggregate study results 79.0% preferred a group meeting for the results to be explained Reason for dissemination: 71.9% identified to understand the benefit of the study to the community	97.7% of participants interested in receiving aggregate study results 92.3% preferred a group meeting for the results to be explained 99.0% of patients preferred a summary in their language Reason for dissemination: 76.6% identified to acknowledge their contribution to the study

* Location of dissemination.

G6PD, glucose-6-phosphate dehydrogenase.

Engagement activities were undertaken at all participating sites at study initiation and throughout the trial period. Engagement was conducted according to local site practices and was not guided by a formalised or standardised framework.

Post-trial result dissemination to study participants was planned to recognise participants’ contribution to the study and share study results. To inform these activities, trial participants were systematically surveyed about their preferences for dissemination at the start of the study.^28^ Of the surveyed participants, overall, 74.6% (601/896) indicated an interest in receiving trial results. Distribution was heterogeneous across study countries, with 33.3% (58/174) expressing interest in Cambodia, 100% (334/334) in Ethiopia, 97.7% (209/214) in Pakistan and 0.0% (0/85) in Indonesia^28^; the lack of interest in Indonesia was potentially explained by the costs of participating in dissemination and how it was communicated to participants. In Ethiopia and Pakistan, most participants (79.0% (264/334) and 92.3% (139/209)) wanted an in-person group meeting, whereas in Cambodia participants preferred individual communication through phone calls (table 1).^28^ Participants in Ethiopia and Cambodia were mainly interested in knowing study results to understand the benefits of the study to the community, whereas in Pakistan, the main motivator among respondents was a wish to be acknowledged for their contribution to the study.^28^

Based on these results, dissemination strategies were developed^28^ and ultimately conducted in four of the seven trial sites: two of the three sites in Cambodia, one site in Ethiopia and one of the two sites in Pakistan (table 2, online supplemental files 1–4). The approach included in-person group meetings in Ethiopia and Pakistan. In Cambodia, participants were recontacted via phone calls during which they were given an option for in-person discussion.^28^ The content of dissemination in respective countries included the study purpose and study results. Study staff also answered questions regarding the treatment of malaria and its prevention. These topics were emphasised differently in each country, with the content focused on an overview of study results in Cambodia, the study and its implications in Ethiopia and thanking patients in Pakistan.

**Table 2:** Summary of dissemination activities

Dissemination characteristic	Cambodia		Ethiopia	Pakistan
Number of study sites where dissemination took place	2 of 3 study sites where 98.6% (217/220) of the Cambodian patients were recruited at these sites^27^		1 of 1 study site.	1 of 2 study sites where 90% (218/240) of the study participants in Pakistan were enrolled^27^
Location of dissemination	Remote and meeting space in the district hospital compound, Phnom Kravanh Pursat Province	Remote and study site within the health centre compound in Siem Pang, Steung Treng Province	Arba Minch University’s comprehensive specialised hospital, located in Arba Minch town, Gamo Zone, Southern Ethiopia Region	Centrally located, easily accessible and spacious café in central Thatta, Sindh Province
Date of dissemination	17–28 February 2025	27–31 March 2025	3 July 2025	23 July 2025
Team	TV, KS, OS, PC and SCS	ME, HV and BA	TS	MAB and NG
Time since last patient follow-up	14 months	15 months	19 months	10 months
Participants	30 participants indicating interest in study results were initially contacted, with the remaining contacted when only 26.7% (8/30) were reached. Eight heard results over the phone and five in person.	28 participants indicating interest in study results were contacted; 12 were reached (42.9%): five heard results over the phone and eight in person.	All participants were contacted, 70.4% (235/334) were reached and 200 attended.	All participants were invited along with two family members; 120 participants contacted, of which 49 (40.8%) attended with 61 family members (total of 110).
Format	Phased dissemination: phone call with option of in-person group meeting. If opted out of in-person meeting, dissemination was conducted over the phone.		Large group meeting with presentation followed by question-and-answer session. Refreshments were provided to the participants.	Large group gathering during which study and its results were explained. Lunch was provided.
Tools	Phone script for phone dissemination (online supplemental file 2), dissemination discussion guide for in-person meeting (online supplemental file 3), handout with brief study explanation and results in Khmer (online supplemental file 1)		Slides prepared for an international audience, but explanation was provided in Amharic.	Handout with brief study explanation and results in Sindhi (online supplemental file 4)
Content of dissemination	Acknowledgement of participant contribution and explanation of study and its results—focus on overview of study results		Acknowledgement of participant contribution and explanation of study and its results—focus on the study and its implications	Acknowledgement of participant contribution and explanation of study and its results—focus on thanking patients

BA, Bipin Adhikari; HV, Hem Vattanak; KS, Kem Sovann; MAG, M. Asim Beg; ME, Mom Ean; NG, Najia Ghanchi; OS, Ou Simvieng; PC, Phal Chanpheakdey; SCS, Sarah Cassidy-Seyoum; TV, Thy Vanda.

### Data collection

An evaluation of dissemination activities occurred at each site where dissemination took place. Methods and results are reported according to COnsolidated criteria for REporting Qualitative (COREQ) guidelines (online supplemental file 5) and with references identifying country (*cm, et* or *pk*) and FGD or interview number (*fgd* or *i*).

#### Cambodia

In Cambodia, in-person group dissemination and its evaluation took place concurrently by the same study staff. This was done to reduce opportunity cost and travel required for patients to attend two sessions and because of the limited number of patients who were reached. Hence, all individuals participating in the dissemination also evaluated it. At the Phnom Kravanh site, a family member of one of the participants also attended the in-person dissemination and evaluation.

Immediately after study results were shared either on the phone or in person, the evaluation was conducted. An over-the-phone satisfaction survey was done by a different member of the study team (online supplemental file 6). Participant responses were recorded on paper, then entered into a Google Form.

Once study results had been shared with participants in person, the study teams (TV, OS, PC, KS, ME and HV) in Phnom Kravanh and Siem Pang facilitated focus group discussions (FGDs, *cm-fgd*) using a discussion guide previously developed (online supplemental file 7). In Phnom Kravanh, an interview was conducted when a participant was unable to join the FGD. In Phnom Kravanh, in addition to the presence of four local study team members, an international team member (SCS) was present and took notes. In Siem Pang, the two local study team members were supported by another international team member who acted both as a facilitator and notetaker (BA). FGDs were audio-recorded with participant permission. Recordings of FGDs were then transcribed and translated in Phnom Penh, Cambodia.

#### Ethiopia

In Ethiopia, an announcement regarding the evaluative discussions was made during the dissemination meeting. The study team had prepared a sign-up sheet for those interested in participating in the evaluation.

A few days after the dissemination itself, FGDs were held at a hotel in Arba Minch town (*et-fgd*). A discussion guide was developed to facilitate the session (online supplemental file 8). In addition to the local FGD facilitator (KG), an international study team member (MM) was present during the FGDs and took notes. FGDs were audio-recorded with participant permission. Recordings of the FGDs were then directly translated by a member of the study team (KG).

#### Pakistan

In Pakistan, the study team visited the homes of dissemination attendees to invite them to participate in the evaluation. The selection process was based on geographic location (to be representative of the main areas from where enrolled participants were based) and proximity to the study site.

The Pakistan study team (AK, SZ, BQ and ZA) conducted the FGDs (*pk-fgd*) facilitated by a discussion guide (online supplemental file 9). The FGDs were organised along sex lines to account for sex dynamics and participant comfort. The FGDs were audio-recorded with participant permission. Recordings of the FGDs were then transcribed and translated by two members of the study team (SZ and BQ).

### Data analysis

**Figure 1:**
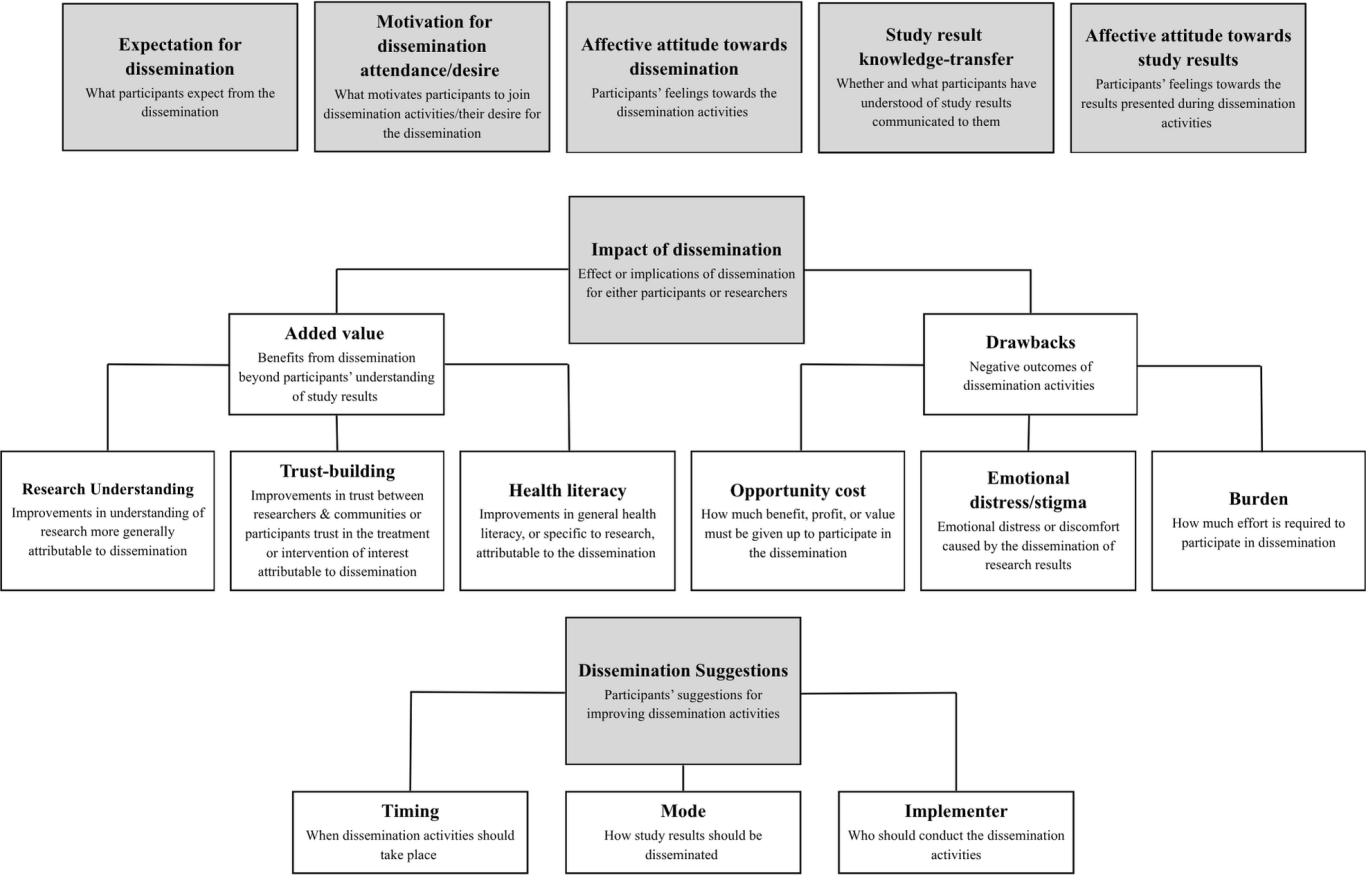
Research dissemination evaluation framework.

All transcripts were reviewed (SCS) after a debrief with all study site teams. A thematic analysis was conducted with an inductive codebook (online supplemental table S1). Transcripts were imported into Dedoose and coded (SCS). Memos were drafted based on themes emerging from a review of code excerpts (individual and combined) and reviewed by respective study site teams (BA, PC, ME, OS, MM, KG, AK, SZ and BQ). Themes included understanding of clinic trials, motivation and expectations for dissemination, knowledge transfer, impact of dissemination, fear and scepticism and dissemination perceptions. These themes informed and were further developed by a derived theoretical framework to evaluate dissemination of research results (figure 1, online supplemental table S2). In addition to study results (themes), the framework was derived from acceptability frameworks^32 33^ and available literature regarding the implementation of dissemination (online supplemental table S2).^10^ Results are presented according to the derived framework components (figure 1), including participants’ dissemination suggestions.

Based on participants’ suggestions, their perceptions regarding dissemination and feasibility considerations, context-informed dissemination recommendations were derived.

The different methodologies used across the different study sites were considered in the analysis of the results, and cross-country comparisons were made with caution. Additionally, while knowledge transfer cannot be determined without a baseline assessment, knowledge regarding the study, its results, research and malaria were explored, and perceived knowledge transfer was assessed. To assess participants’ knowledge and perceived knowledge transfer, the differences in content presented in the different study countries were considered.

### Patient and public involvement

Patients or the public were not involved in the design, conduct, reporting or dissemination plans of our research.

## Results

### Participant characteristics

In Cambodia, the 25 respondents who participated in the dissemination were included in the evaluation. One FGD with five respondents (four patients and one family member) was conducted in Phnom Kravanh (*cm-fgd1*), while two FGDs with four and three respondents (*cm-fgd2,3*) were conducted in Siem Pang. An interview was also conducted with one participant in Phnom Kravanh (*cm-i1*). In Ethiopia, of the 200 participants who attended the meeting, 35 signed up and 34 ultimately participated. Six FGDs were conducted (*et-fgd1-6*), with 5–7 respondents in each (total of 34 respondents). In Pakistan, of the 35 who were invited, 23 participated in four FGDs (*pk-fgd1-4*). There were two groups of six male respondents and two groups of female respondents, with six and five respondents, respectively.

Across the three study site countries, there were a total of 82 dissemination evaluation participants, of whom 25 (30.5%) were enrolled in Cambodia, 34 (41.5%) in Ethiopia and 23 (28.0%) in Pakistan. Of the 25 participants in Cambodia, 12 evaluated the dissemination that they had received over the phone through a phone survey (table 3). The median age of in-person evaluations was 33 years in Cambodia (excluding patient family member), 26 in Ethiopia and 28 in Pakistan (table 3). The age distribution in the sample was similar to the overall study population, with the median age in Cambodia, Ethiopia and Pakistan being 26, 23 and 31, respectively. Also comparable to the overall study population, in Cambodia and Ethiopia, females made up 33% (4/12) and 35% (12/34) of in-person participants, respectively (females making up 10.0% of total participants in Cambodia and 44.7% in Ethiopia). However, in Pakistan, females accounted for 50% (11/22) of in-person participants compared with 26.3% of total participants enrolled. In Ethiopia, 82.3% (28/34) of in-person participants had at least a high school education, in contrast to 21.7% (5/23) in Pakistan and 58.2% (7/12) in Cambodia.

**Table 3:** Socio-demographic information for dissemination evaluation participants in Cambodia, Ethiopia and Pakistan

Country*	Study site	Participants	Evaluation type	Median age†	Sex	Education level	Occupation
Cambodia	Phnom Kravanh	5	In-person FGD/interview	40 (32–52)	2 women 3 men	Grade 4: 2 High school: 1 Unknown: 2	Farmer (2), teacher (1), unknown (2)
Cambodia	Siem Pang	7	In-person FGD	27 (22–36)	2 women 5 men	No education: 1 High school: 6	Teacher (2), NGO employee (2), soldier (2) and farmer (1)
Cambodia	All sites	12	In-person FGD	31 (18–52)	4 women 8 men	No education: 1 Grade 4: 2 High school: 7 Unknown: 2	Farmer (3), teacher (3), soldier (2), NGO employee (2) and unknown (2)
Cambodia	Phnom Kravanh	8	Phone Survey	24 (20–36)	1 woman 7 men	Primary school: 1 Grades 8–11: 5 High school: 1 Unknown: 1	Farmer (5), soldier (2) and unknown (1)
Cambodia	Siem Pang	4	Phone Survey	30 (26–30)	4 men	High school: 4	Soldiers (4)
Cambodia	All sites	12	Phone Survey	26 (20–36)	1 woman 11 men	Primary school: 1 Grades 8–11: 5 High school: 5 Unknown: 1	Farmer (5), soldier (6) and unknown (1)
Cambodia	Overall	24	Overall	29.5 (20–52)	5 women 19 men	No education: 1 Grade 4: 2 Primary school: 1 Grades 8–11: 5 High school: 12 Unknown: 3	Farmer (8), teacher (3), soldier (8), NGO employee (2) and unknown (3)
Ethiopia	Arba Minch	34	In-person FGD	26 (18–61)	12 women 22 men	No education: 1 Primary school: 5 High school: 11 Diploma: 6 Bachelor’s: 10 Master’s: 1	Civil servant (4), driver (1), housewife (1), professor (1), teacher (2), merchant (1), motorbike driver (1), public transport conductor (1), retired (1), road constructor (1), security (1), self-employed (2), university students (8), unemployed (8) and unknown (1)
Pakistan	Thatta	23	In-person FGD	28 (19–48)	11 women 12 men	No education: 8 Grades 5–8: 6 Grades 9–11: 4 High school: 1 Bachelor’s: 1 Unknown: 4	Civil hospital trainee (1), district commissioner office (2), drycleaner (1), hotel staff (1), housewife (5), ironer (1), labourer (1), maid (2), mechanic (1), mobile shop worker (1), student (1), tailor (2), unemployed (1) and unknown (3)

* Cambodia socio-demographic data excludes information on patient/participant family member.

† Median age is followed by age range in parentheses.

FGD, focus group discussion; NGO, non-government organisation.

Results are presented according to the framework components, starting with expectation and motivation for dissemination, followed by knowledge transfer, impact of dissemination, affective attitudes towards dissemination and participants’ dissemination suggestions and concluding with derived context-specific dissemination recommendations.

### Expectation and motivation for dissemination

Respondents in Cambodia and Ethiopia expressed commitment to participating in dissemination activities, demonstrating key motivation factors for their attendance. The latter included a wish to know both individual (*cm-fgd2* and *et-fgd4*) and aggregate (*cm-fgd2* and *et-fgd4,1*) results because they had given their blood for the study (*et-fgd4,6*), satisfaction with their trial and treatment experience (*cm-fgd3*) and being able to “to see the result of the study together” (*et-fgd3*), as a community.

Thus, the importance of patients’ personal recovery from malaria serves to motivate patients’ participation in dissemination activities to understand how this affects their personal health.

*We have been sick for months. (We wanted to know) what the possible effects could be and whether it can be completely cured or might recur*. cm-fgd2

Such motivations are in line with some patients’ expectations for the dissemination in Ethiopia and Cambodia (*et-fgd4* and *cm-fgd2*). Instead of receiving aggregate results, some patients expected to receive personal results individually in person or by email (*et-fgd4*). One participant expressed it simply: “I expected my own result because they had told me that I would hear the result”. Justifying why one participant wanted to receive their own results was so that “[…] we can know whether relapse occurred on me…whether it has caused haemolysis on me…when it has removed the parasites from my body” (*et-fgd4*).

### Knowledge and perceived indications of knowledge transfer

The level of knowledge identified differed between countries. In Ethiopia, respondents had the greatest depth of understanding of the study, its purpose, its results, as well as knowledge of malaria itself and its prevention and treatment, notwithstanding varying levels of understanding across the FGDs (*et-fdg1-6*). In Cambodia, respondents had a degree of understanding of the study’s objective and its results in addition to malaria prevention and treatment knowledge (*cm-fgd1-3* and *i1*). In contrast, respondents in Pakistan were only able to identify two treatment options and describe malaria prevention strategies when given the opportunity (*pk-fgd3,4*).

In Cambodia and Pakistan, some participants could not share what they had learnt—some of them citing that they had forgotten (*cm-fgd1*), that they had arrived at the meeting late (*pk-fgd4*) or did not understand the results presented to them (*pk-fgd2*).

Among respondents with a better understanding of the study and its results, there was an awareness that the study tested the effectiveness of different treatments (*cm-fgd1,2* and *et-fgd2-5*) and identified the most effective treatment (*cm-fgd2* and *et-fgd1,3,4*). Some of the respondents also described the limited nature of the side effects associated with the treatments (*cm-fgd2* and *et-fgd1,3-5*); one participant in Ethiopia understood that with an increase in the dose of the treatment, there may be more side effects (*et-fgd4*), whereas another described the need for G6PD testing (*et-fgd2*), risk of haemolysis (*cm-fgd2, et-fgd1*) and, if it occurred, that they could get a blood transfusion (*et-fgd1*). Participants attributed some of this knowledge to the dissemination:

*Comparative analysis was made on all types of medicine in terms of their side effects and effectiveness to remove the parasites…so the results of the study have helped us to have a better understanding…. The results shared openly with us have helped to increase our awareness*. et-fgd3

*R: You told us about the effectiveness of the 1-day and 7-day medications, which could heal faster. In short, those countries applied the same treatment methods*.

*R1: I would definitely choose the 1-day medication.* cm-fgd1

While knowledge transfer did take place through dissemination, a lack of clarity and misunderstandings regarding the study itself and malaria treatment remained (*cm-fgd1* and *et-fgd5,6*). For instance, in Ethiopia and Cambodia (*cm-fgd3*), respondents were sometimes unclear about why everyone in the trial was not given the same treatment for the same disease (*et-fgd6*).

*For the same disease, the same type of medicine should be given…however, they gave us different medicine…some patients received smaller while others received many*… et-fgd6

This lack of understanding is also illustrative of a broader blurring of lines between care received during a clinical trial and routine care—which the dissemination did not rectify (*et-fgd3,5,6 and pk-fgd1,2*). For instance, in Pakistan and Ethiopia, questions arose as to why treatment was not provided to children (*et-fgd6* and *pk-fgd1*). Additionally, based on the quality of care provided by the clinical trial staff, there was an expectation that free treatment would be provided for other diseases beyond the trial (*et-fgd3,5* and *6pk-fgd2*). The blurred line between routine and trial care is best described by respondents in Pakistan, stating: “P6: This system should continue, this centre. P1: Yes, when it was closed in between, we were very worried, so this system should continue” (*pk-fgd*1).

### Impact of dissemination

In Cambodia and Pakistan, dissemination was associated with an expectation among some participants that they could continue to access the trial treatment after trial completion (*cm-fgd1, l1* and *pk-fgd2,3*). There were several positive impacts in all three countries, reflecting the dissemination’s added value beyond facilitating an understanding of the study purpose and its results. These included an improvement in (i) participants’ malaria health literacy (*cm-fgd-2,3, i1, et-fgd1,3-5* and *pk-fgd3*) and (ii) in their research/trial understanding (*cm-fgd2,3, et-fgd1,2,3,4,*6 and *pk-fgd2*) in all three countries, (iii) building trust in clinical trials, new treatments and researchers (c*m-fgd2,3, et-fgd1-4* and *pk-fgd1,2*) and (iv) gaining an understanding of being a part of something bigger in Cambodia and Ethiopia (*cm-fgd3, i1* and *et-fgd1-4*).

#### Added value: health literacy

A key positive impact of the dissemination process, which was identified in all three countries, was an improvement in malaria health literacy (*cm-fgd2,3, i1, et-fgd1,3-5* and *pk-fgd3,4*). Trial result dissemination was an opportunity for study staff to conduct health education pertaining to malaria (*cm-fgd2, i1, et-1-6* and *pk-fgd2,3*), but equally, it was an opportunity for patients to ask questions regarding malaria and their experience with treatment in the trial and outside of it (*cm-fgd2, i1, et-1-6* and *pk-fgd2,3*). A question that arose in Cambodia and Ethiopia was regarding recurrent episodes of malaria and treatment effectiveness. As a result of the trial staff’s explanations regarding relapse and the need to maintain prevention measures, patients had a clearer understanding of this and what they needed to do to remain malaria-free (*cm-fgd2, i1* and et*-fgd3,*5).

#### Added value: research understanding

Understanding of clinical trials or research in general varied between Cambodia, Ethiopia and Pakistan. Participants in Ethiopia demonstrated the most understanding of the purpose of clinical trials and what they entail (*et-fgd1,2,3,4,6*). Respondents understood that they were participating in research with a larger goal than their personal treatment (*et-fgd1,3,4,6*). This notion of self-sacrifice for the greater public good was expressed by more than one respondent in Ethiopia (*et-fgd3,6*).

*To your surprise, our consent was asked first…We started voluntarily…**Even I am happy to sacrifice myself and save other people…I should save my children and the next generation**.* et-fgd4

In Cambodia and Pakistan, some understanding of this objective emerged after further explanations during the focus group discussions (*cm-fgd2,3* and *pk-fgd2*).

#### Added value: trust-building

Dissemination built trust in research and researchers among participants by offering a space for patients to share their experiences (*cm-fgd2,3* and *et-fgd1*), removing stigma associated with study participation in Ethiopia (*et-fgd1-4*) and, in Pakistan, by showing patients that they are valued (*pk-fgd1,2*).

Bringing patients together for the dissemination was also an opportunity for them to share their experiences with malaria, its treatment and other illnesses (*cm-fgd2,3* and *et-fgd1*). Sharing these experiences allowed for more trust-building, motivating future participation in future clinical trials or research (*et-fgd1*). Another element from the dissemination that added to the trust-building and understanding of the clinical trial in Ethiopia was reducing misunderstanding pertaining to study participation, quelling rumours and stigma surrounding the trial (*et-fgd1-4*).

There were misconceptions among participants and community members about reimbursements for costs incurred and care received as part of the trial, as well as the several blood draws (*et-fgd1-6*). The first of the misconceptions was that the study used their blood for other purposes, including selling it (*et-fgd3,5,6*). The second was that participants were providing their blood for membership in a religious cult termed the ‘666’ (*et-fgd2-6*). Other perceptions included that participants were being experimented on and that the treatment provided as part of the trial caused serious and potentially fatal outcomes (*et-fgd3,4*). Participants identified how beneficial the dissemination activities were in clearing up any scepticism, fear or mistrust (*et-fgd1-4*).

*There was a rumour that the medicine we have been taking would cause heart failure in the long run. Therefore, from the previous presentation, I understood that these rumours are all lies*. et-fgd3

Lastly, within the cultural context of Pakistan, gestures such as being invited for lunch and receiving attentive hospitality made patients feel valued and helped build trust and strengthened their emotional connection to the information shared during the sessions (*pk-fgd1,2*).

#### Added value: feeling a part of something bigger

Another positive impact of the dissemination activities in Cambodia and Ethiopia was that participants developed awareness and understanding that they were a part of something larger, both in their own country and across the world (*cm-fgd3, i1* and *et-fgd1-4*). Participants became more aware that malaria affected many in their community and other countries (*c-i1* and *et-fgd1,3,4*). As one participant in Ethiopia stated, “I thought it was only me who had been making the treatment. However, I was surprised when I saw a large number of people in the hall during the presentation. I thought I was the only person who had the disease” (*et-fgd1*). A participant in Cambodia noted that “We did not know that before. We thought it was only Cambodia” (*cm-fgd3*). Understanding the scale of the study and their place in it led participants to be more appreciative of the study and their individual contribution (*et-fgd2,3*).

### Affective attitudes: dissemination satisfaction

Overall, participants in all three countries expressed satisfaction with the dissemination activities (*pk-fgd1,2,3,4, cm-fgd1,2,3, i1* and *et-fgd1-6*). This satisfaction came from being able to learn and understand the study results (*cm-fgd1-3* and *et-fgd1,6*) despite not learning about their individual results (*et-fgd4*), understanding their contribution to the larger trial (*et-fgd1,4*), having enough time to ask questions (*et-fgd1,3,4,5*), sharing their treatment experiences (*cm-fgd2* and *et-fgd1*), being able to share results with others in their communities (*cm-fgd2*), learning useful information (*pk-fgd2*) and feeling valued and respected (*pk-fgd1,2*). In Ethiopia and Pakistan, participants expressed a sense of gratitude (*et-fgd1* and *pk-fgd1*), while in Ethiopia and Cambodia, participants expressed their satisfaction with the clear explanations they received (*cm-fgd3,i1* and *et-fgd1-6*). Participants in Ethiopia specified that the explanations in Amharic were still clear and considerate of varying levels of education and age groups, despite the slides being in English and some technical language being used (*et-fgd1-4*).

In Cambodia, participants were also given an option to hear study results over the phone. Those who chose to receive results over the phone were asked to complete a satisfaction survey at the end of the call, and of the 12 participants who participated in dissemination over the phone, 11 were very satisfied and one was satisfied with how the study results were explained to them.

In the in-person evaluations across the settings, satisfaction with the dissemination was often overshadowed, especially in Cambodia and Pakistan, by general trial satisfaction and the quality of care received as part of the trial (*cm-fgd3, i1, et-fgd4* and *pk-fgd3*).

***I was waiting eagerly for the result****…**the way (name removed) treated us was so wonderful…he has been calling us to remember the appointment**…I expected my own result because they have told me that I would hear the result…however, a summarised report was presented…anyway it was good*. et-fgd4

Q: Please share your experience

*R: It was very good. My health had become very bad, but when I came here, I got excellent treatment.* pk-fgd3

Participants’ impression of the handouts provided in Cambodia and Pakistan is summarised in table 4.

**Table 4:** Summary plain language and feedback on the visual study result handout

Feedback type	Feedback	Country	Source
Use	All participants were provided the handout during the dissemination/dissemination evaluation and took it home	Cambodia	Study team
	Some participants reported not receiving the handout, not having read it or not taking it home with them	Pakistan	*pk-fgd3,4*
Readability	Some participants were able to read the handout	Cambodia	*cm-fgd2,3*
	Participants indicated the visuals were useful, while not being literate enough to read the text	Pakistan	*pk-fgd1,3,4*
Understanding	Some could understand the handouts, whereas one participant indicated needing an explanation from the study staff to fully understand what the handout communicated	Cambodia	*cm-fgd2,3*
	Participants could understand the main messages from the visuals alone	Pakistan	*pk-fgd1,3,4*
	Participants could understand which treatment was the most effective by looking at the handout	Pakistan	*pk-fgd1*
Satisfaction	Participants found the handout good and informative	Pakistan	*pk-fgd1*

### Participant suggestions

While the dissemination activities were well accepted by participants, participants in Cambodia and Ethiopia had several suggestions regarding the content and how to improve the experience and the accessibility of the information. These suggestions are detailed in online supplemental table S4 in the online supplemental materials.

A recommendation that emerged in all three countries was ways for study results to be communicated to the wider community beyond the trial participants (*cm-fgd3, et-fgd1-6* and *pk-fgd2,4*).

R2: Tell people in your neighbourhood, I will tell people in mine. In this way, the message will spread, and then they will pass it on further.

*R1: Tell one another.* pk-fgd2

Rationale for this wider community dissemination included removing the stigma around the trial in Ethiopia (*et-fgd5*), wanting to share with others about the availability of free and effective treatment (*cm-fgd2,3, et-fgd2,4* and *pk-fgd2*) and improving their malaria transmission, treatment and prevention knowledge (*et-fgd2,5,6*). Suggested methods for public dissemination included community meetings in Cambodia and Ethiopia (*cm-fgd3* and *et-fgd4,5*) and sharing through text messages, WhatsApp or Telegram in Ethiopia and Pakistan (*et-fgd2,3,5* and *pkfgd2,4*). In Cambodia, participants also suggested dissemination through village chiefs (*cm-fgd1*). In Ethiopia, other suggestions included disseminating through TV programmes (*et-fgd1,2,6*), radio (*et-fgd1,5,4*), social media (*et-fgd1,3,4*), flyers (*et-fgd3,4,5*) and healthcare provider health education (*et-fgd1,2*) at public and private facilities (*et-fgd1*).

Furthermore, participants identified their potential role in disseminating trial information to their communities (*cm-fgd2, et-fgd1-6* and *pk-fgd1,2*). This was especially the case in Ethiopia where some participants identified sharing their experience as a responsibility (*et-fgd5,6*). In Pakistan, they mentioned using the handout provided to them to share results with their community (*pk-fgd2*).

Some respondents in Ethiopia recognised that the treatments tested as part of the trial were not yet part of the policy or approved—which would be a consideration as to whether to disseminate to the public (*et-fgd1,6*).

*It can also be published, but only well-educated people can access it. We know that Tafenoquine and Primaquine for 7 days **are not legalised in the healthcare system. If it is required to be publicised, all community channels should be used***. et-fgd1

### Recommendations

Based on participant suggestions, perceptions of dissemination and feasibility considerations, context-specific recommendations are reported in table 5.

**Table 5:** Context-informed recommendations derived from participants’ suggestions presented according to the SHOW RESPECT framework for planning dissemination^66^

Dissemination recommendations	Planning consideration type	Recommendation source
Dissemination should include a refresher on the study purpose and study progress updates during treatment follow-up.	**S**upporting and preparing trial participants to receive results **T**iming—when should results be communicated?	Derived from results in framework components: expectations for dissemination and participant suggestions
Main post-trial completion dissemination should be conducted in person and in a group format.	**HO**w will the communication tool(s) reach participants?	Derived from results in framework components: participant suggestions and affective attitudes and dissemination satisfaction
Alternative dissemination methods should be employed for those unable to attend in person (eg, phone calls and home visits).	**HO**w will the communication tool(s) reach participants? **C**ommunication tools—which ones will you use?	Derived from results in framework components: participant suggestions and impact of dissemination
A plain language summary of the study and its results (handout) should be provided to participants both in person and electronically.	**HO**w will the communication tool(s) reach participants? **C**ommunication tools—which ones will you use?	Derived from results in framework components: participant suggestions and affective attitudes: dissemination satisfaction
Participants’ language needs should be identified during the trial.	How will the communication tool(s) reach participants? **C**ommunication tools— which ones will you use?	Derived from results in framework components: participant suggestions and affective attitudes: dissemination satisfaction
All dissemination activities and content should be in the local language(s).	**Who** are the trial participants?	Derived from results in framework components: participant suggestions, affective attitudes: dissemination satisfaction and impact of dissemination
Participant preference for level of detail of shared study results should be determined during the trial.	**Who** are the trial participants?	Derived from results in framework components: knowledge and perceived indications of knowledge transfer and affective attitudes and dissemination satisfaction
In addition to an overview of the study and its results, dissemination of content should also include a brief on the current malaria situation and the availability of the trial treatments.	**RE**sults—what do they show **Who** are the trial participants?	Derived from results in framework component: participant suggestions
Dissemination of individual study results should be considered.	**Who** are the trial participants?	Derived from results in framework components: expectations for dissemination and participant suggestions
Public dissemination should be considered prior to research translation into policy.	**HO**w will the communication tool(s) reach participants? **Who** are the trial participants?	Derived from results in framework components: participant suggestions and impact of dissemination

## Discussion

Respondents were motivated to attend dissemination activities to receive study results with the potential to inform their personal health. There was also some expectation of receiving individual results rather than aggregate trial results. Knowledge and indications of knowledge transfer varied by country, potentially reflecting differences in dissemination methodology. There were some misunderstandings regarding the trial, its treatment and results among participants in all countries—most notably a blurring of lines between the care provided during the trial and during routine care. Dissemination impact included the improvement of participants’ malaria health literacy and general understanding of research, as well as building trust in the trial and research generally. Overall, participants were satisfied with the dissemination because they gained an understanding of the trial, its results and malaria prevention mechanisms and because they could fully appreciate their contribution to the study and feel a part of something larger and highly valued. Participants did, however, offer some recommendations regarding accessibility and content and emphasised the importance of in-person dissemination and public dissemination.

Our findings on motivations for wanting to hear about study results are in line with motivations expressed in our survey during the trial in Ethiopia and Pakistan.^28^ Ethiopian participants valued clinical trials as an important public good; hence, they wanted to hear the results as a community, whereas participants in Pakistan identified wanting to feel valued as the main motivator for trial result dissemination. Similar to research participants in other contexts,^10 34^ in Ethiopia and Cambodia, motivations for dissemination were also largely driven by wanting to know the results of the study in relation to their health and well-being, expressing a desire for individual results.

Although including individual results was beyond the scope of our dissemination efforts, almost a quarter of participants surveyed as part of the clinical trial wanted to know their individual results,^28^ and this was an expectation that was reported among Ethiopian participants in the evaluation as well. Such a desire for individual results is in line with surveys conducted among participants in cancer, diabetes and genetics research.^35–37^ However, there may be feasibility and logistical challenges with providing participants with individual results post-trial completion. The need to include individual results as part of post-trial dissemination could be mitigated by clear and consistent communication regarding individual tests and their results during trial visits. Additionally, during post-trial dissemination, there may be opportunities to better relate aggregate results to participants’ individual experiences, highlighting how individual patients fit within these aggregated results and thereby better addressing patients’ motivation of knowing how the results inform their own health and well-being.

Knowledge transfer can be challenging since this requires the translation of complicated scientific findings into key information that participants can understand.^38^ Factors that could have affected the varying extent of knowledge transfer across study sites included participants’ literacy and education level, motivation for the dissemination^28^ and how and the extent to which the trial results were communicated to participants. There was a contrast in the education levels between study site participants, with education levels higher among participants in Ethiopia. The variation was reflected in the level of detail provided to patients and their understanding. The study findings, therefore, suggest that there is a need to better determine the literacy and education level of clinical trial participants and tailor dissemination better.

A misconception that was not addressed by dissemination activities was the role of research or clinical trials relative to routine care. Some participants in Pakistan and Ethiopia described, expected and recommended provision of health services beyond malaria care as part of the trial. As has been found in other contexts,^39–41^ given the quality of care received during the trial, participants wanted this care to continue and expand to include more of their community members. These blurred lines between trial and routine care are partially attributable to how research sites and health facilities are physically integrated and how well these research sites are embedded into communities and participants’ health-seeking pathways, given their longstanding presence in the community conducting different research projects. Such an understanding likely reflects the need for clearer communication during the informed consent process, taking into consideration that this communication needs to be balanced against fuelling patients’ fears of being experimented on.

These blurred lines also bring into question the ethical responsibility clinical trial teams and researchers have in post-trial access and care.^42^ There are conflicting views on the need or lack thereof to provide post-trial access, such as arranging access to investigational interventions.^42^ However, proponents of post-trial access ground their support for it in equity and avoiding exploitation, especially in the context of LMICs, where participants might not have access to quality or required care outside of the clinical trial context.^42–44^ For some, post-trial access should go beyond arranging access to the clinical trial intervention to include responsible transitioning to needed healthcare.^44–46^ Integrating post-trial care as a topic of discussion during the informed consent process would contribute to patients’ understanding of the trial and help distinguish between the trial and routine services.

Participants were satisfied overall with both the dissemination process and the explanations provided. Although social desirability bias could have affected reported satisfaction, participants offered some suggestions to improve dissemination activities, showing openness to report critically. Similar findings pertaining to dissemination satisfaction have been reported from Zambia and Uganda, where dissemination activities allowed participants to feel gratitude, valued and proud.^47 48^ Participants in Cambodia and Pakistan also expressed satisfaction with the visual and lay-language informational handouts, supporting understanding, as they have previously shown to do.^49–51^ In addition to helping participants understand the study and its results, the dissemination also improved participants’ malaria health literacy and understanding of research while increasing trust in research and researchers.^10^ Another dissemination benefit was making participants feel they were a part of something larger, learning they were not the only ones affected by malaria in their community or globally while allowing them to share their experiences and contributing to building trust.^52^

Study participants shared experiences and recommendations, which informed the context-specific recommendations outlined in table 4 that could be applied in similar resource-limited settings. Among these, a key recommendation was to disseminate results beyond the study participants. Given the importance to participants of sharing the study results and experiences, community dissemination should be considered by research teams. Participants identified their role in this broader dissemination, expressing a desire to share their knowledge and experiences—an exercise which clinical trial managers had identified elsewhere as a means of expanding the benefit of dissemination.^16^ Although there are benefits to trust-building and improving the acceptability of interventions,^16 53^ a consideration of broader community dissemination is whether research results have been applied to current practices. If community dissemination is to take place, the availability of the intervention or treatment of interest would need to be clearly articulated to avoid any community disappointment.

Slow policy change processes that incorporate positive research findings can be an important barrier to participant dissemination. As in our study, dissemination can make patients aware of better treatment options that may not be routinely available at the point of dissemination. This makes communication of study results more complicated, potentially deterring investigators from disseminating results; however, research teams can work with national programmes on appropriate messaging, while awareness of results might also empower patients to advocate for the uptake of new and better treatment options.^54^

This study has several limitations. First, contacting trial participants was challenging in all three study countries. Difficulties were most pronounced in Cambodia, where most participants were not reachable, limiting the number of patients who could participate in the dissemination activities and their evaluation. Second, different dissemination evaluation methods were used in the different study countries, making cross-country comparisons difficult. Third, the dissemination and its evaluation took place at the same time by the same study team in Cambodia, and this likely introduced bias and limited participants’ openness to share potentially critical feedback and favouring expression of satisfaction with the dissemination. Fourth, in Ethiopia and Pakistan, participants who agreed to participate in the evaluation (FGDs) may have been those most interested in the study and understanding of it, influencing the results regarding level of knowledge about the study and its results. Fifth, a pre-dissemination baseline assessment of knowledge was not conducted, limiting conclusions regarding the effectiveness of the dissemination in transferring knowledge. Sixth, in Pakistan, the format of the FGDs often involved revisiting and summarising the study results, which constrained our ability to differentiate participants’ pre-existing understanding from responses informed by the summary. Seventh, further research is needed to explore participants’ expectation of receiving individual results and how that could be implemented. Lastly, this study evaluated the dissemination experience from a participant standpoint, excluding the perspectives of family members, the broader community, country partners and the site study teams.

## Conclusion

Our findings contribute to the very limited data on dissemination of trial results in LMICs, providing context-informed recommendations that can be applied and adapted to other resource-limited settings. Equally, our recommendations underscore the importance of consulting participants about their preferences for dissemination and tailoring activities accordingly, as well as potentially providing individual results and implementing broader community dissemination.

## Supplementary material

10.1136/bmjopen-2026-119698 Supplementary file 1

## Data Availability

Data are available upon reasonable request.
