# Supplementary material for: Dissemination of trial results to study participants in Cambodia, Ethiopia and Pakistan: a qualitative study
Source: BMJ Open. 2026 Aug 10;16(8):e119698. doi: 10.1136/bmjopen-2026-119698 (PMC13475131; doi:10.1136/bmjopen-2026-119698)
Supplement: Supplementary file 1 [file bmjopen-16-8-s001.pdf]

Supplementary Materials for paper titled,

"Evaluation of a malaria clinical trial result dissemination to participants in Cambodia, Ethiopia, and Pakistan: A Qualitative Study."

File S1: Trial handout provided to participants in Cambodia

File S2: Dissemination phone script for participants in Cambodia

File S3: Dissemination content guide for study team in Cambodia

File S4: Trial handout provided to participants in Pakistan

File S5: COREQ Guideline Checklist

File S6: Phone dissemination satisfaction survey for participants in Cambodia

File S7: Evaluation focus group discussion guide for study team in Cambodia

File S8: Evaluation focus group discussion guide for study team in Ethiopia

File S9: Evaluation focus group discussion guide for study team in Pakistan

Table S1: Joint analysis codebook

Table S2: Development of components of the Research Dissemination Evaluation Framework

Table S3: Details of study ethical approval

Table S4: Specific suggestions for dissemination from participants

File S1: Trial handout provided to participants in Cambodia, English and Khmer version

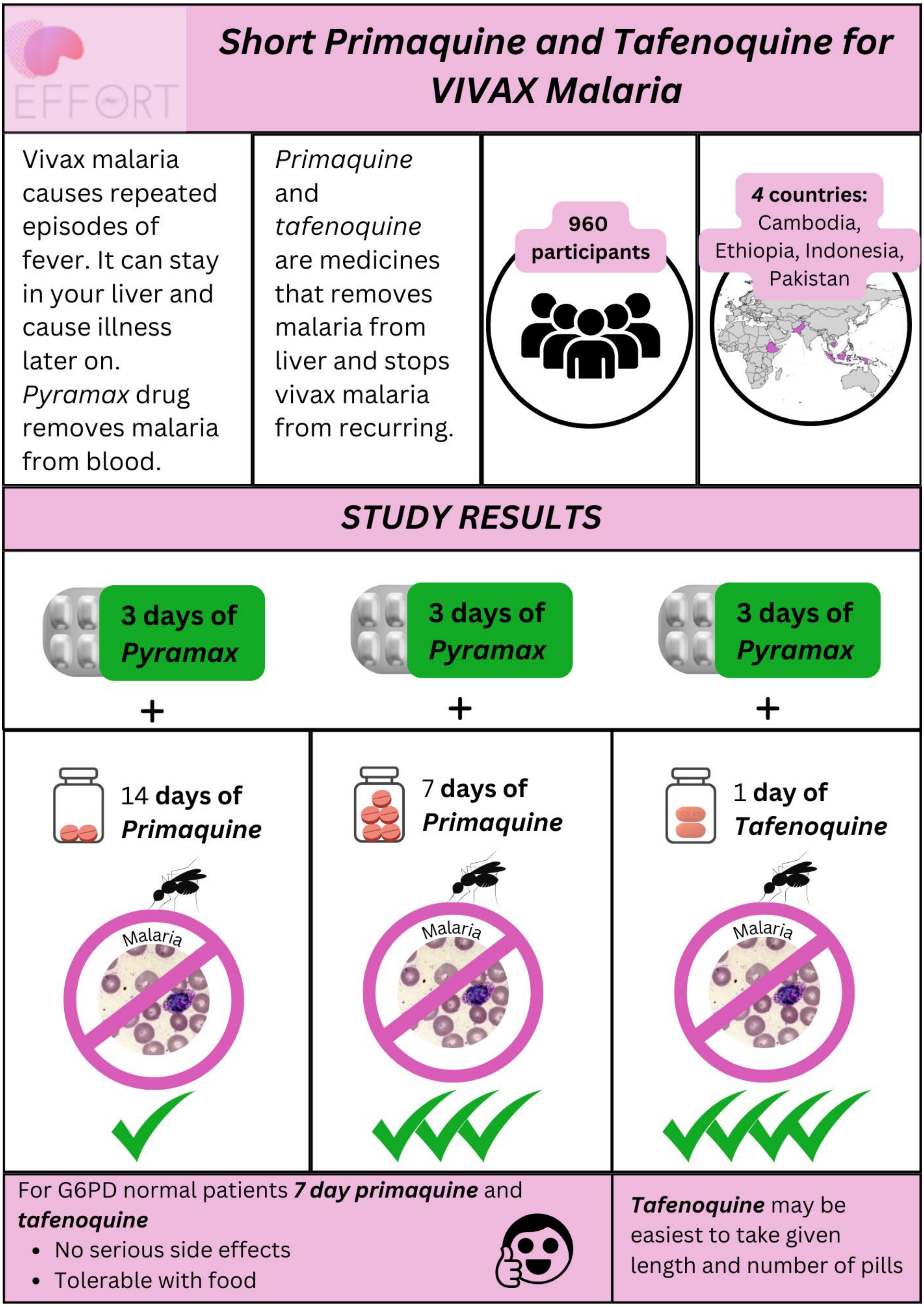

ការប្រើប្រាស់ថ្នាំប្រីម៉ាគ្វីន *(Primaquine)* និងតាហ្វេនូគ្វីន *(Tafenoquine)* ដើម្បីព្យាបាលជម្ងឺគ្រុនចាញ់ *(Vivax Malaria)* នៅក្នុងរយៈពេលខ្លី

Vជំងឺគ្រុនចាញ់វីវ៉ាក់ (*Vivax*) បណ្តាលឱ្យមានគ្រុនក្តៅម្តងហើយម្តងទៀត។ វាអាចស្ថិតនៅក្នុងថ្លើមរបស់អ្នក និងបង្កជាជំងឺនៅពេលក្រោយទៀត។.

ថ្នាំធីរ៉ាម៉ាក់ *(**Pyramax**)*: ជាថ្នាំដែលជួយលុបបំបាត់ជម្ងឺគ្រុនចាញ់ចេញពីឈាម។.

ថ្នាំប្រីម៉ាគ្វីន *(**Primaquine**)* និង**តាហ្វេនូគ្វីន** *(**Tafenoquine**)*: ជាថ្នាំដែលលុបបំបាត់ជម្ងឺគ្រុនចាញ់ចេញពីថ្លើម និងបញ្ឈប់ការកើតឡើងវិញនៃជម្ងឺគ្រុនចាញ់ *(Vivax Malaria)*។

ចំនួនអ្នកចូលរួម៖
960

***4* ប្រទេស៖**
កម្ពុជា, អេត្យូពី, ឥណ្ឌូណេស៊ី, ប៉ាគីស្ថាន

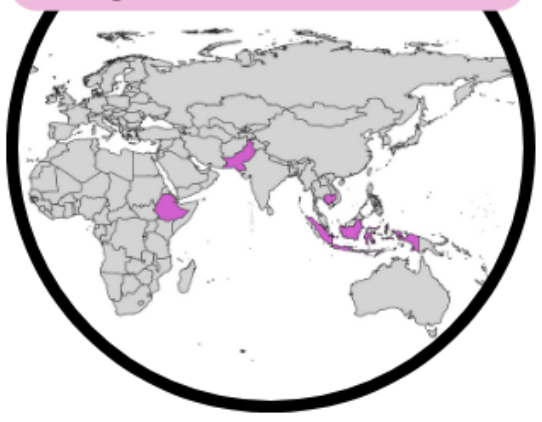

លទ្ធផលនៃការសិក្សា

**ថ្នាំធីរ៉ាម៉ាក់ (Pyramax) 3 ថ្ងៃ** +	**ថ្នាំធីរ៉ាម៉ាក់ (Pyramax) 3 ថ្ងៃ** +	**ថ្នាំធីរ៉ាម៉ាក់ (Pyramax) 3 ថ្ងៃ** +
**ថ្នាំប្រីម៉ាគ្វីន** (***Primaquine***) 14 ថ្ងៃ	**ថ្នាំប្រីម៉ាគ្វីន** (***Primaquine***) 7 ថ្ងៃ	**ថ្នាំតាហ្វេនូគ្វីន** (***Tafenoquine***) 1 ថ្ងៃ
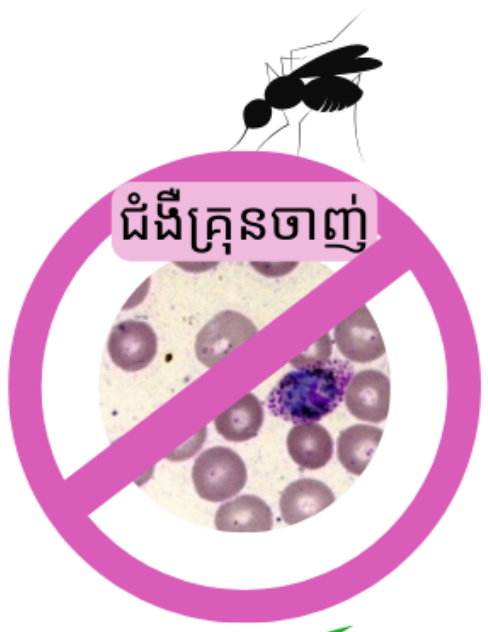	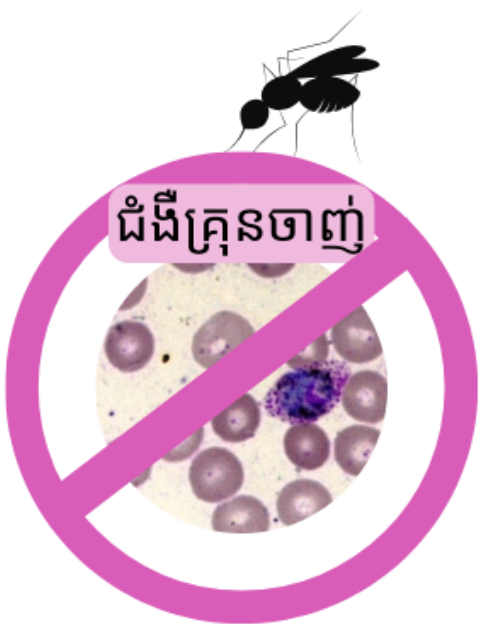	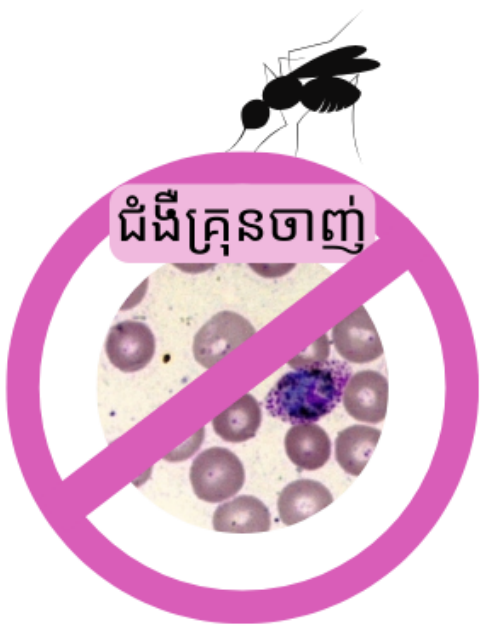

សម្រាប់អ្នកជម្ងឺដែលមានជាលិកាឈាម G6PD ធម្មតា, **ថ្នាំប្រីម៉ាគ្វីន** (***Primaquine***) **7 ថ្ងៃ** និង**តាហ្វេនូគ្វីន** (***Tafenoquine***)គឺមានប្រសិទ្ធិភាព។
- គ្មានផលប៉ះពាល់ធ្ងន់ធ្ងរ។
- អាចទទួលយកបានពេលញុំាអាហារ។

ថ្នាំតាហ្វេនូគ្វីន (***Tafenoquine***) អាចជាផ្លូវល្អបំផុតសម្រាប់ការប្រើប្រាស់ ដោយសារតែវាមានចំនួនថ្នាំតិច និងប្រើប្រាស់រយៈពេលខ្លី។

File S2: Dissemination phone script for participants in Cambodia

EFFORT Patient Dissemination Phone Script

Hello _____,

This is ________ from MORU, the study you were a part of that provided treatment for your malaria. Do you remember participating in this study?

Answer Options 1: I do not remember.

Response: Remember you came for malaria treatment to [Phnom Kravanh district hospital OR Siem Pang health center] and you had to come back for us to check you often.

Answer Option 2: Yes, I remember.

During your enrolment you indicated that you wanted to receive the study results once the study was completed. We have now completed the study and can share the results with you. Would you prefer to get the results over the phone, or would you like to come to ________ health center to discuss the results with other patients? We will provide refreshments at the health center and cover your transportation cost to come to the health center if you chose that option.

Answer Option 1: I would like to come to the health center.

Response: Great. We are available for briefings in person on Monday, February 24, or Tuesday or Wednesday morning. When would you be available?

Answer Option 2: I would prefer to hear the results over the phone.

Response: Okay, then we will now share the results of the study. Please stop me at any point if you have questions.

As part of the study, you could have received 3 treatment options:

1. treatment [with Pyramax and high dose primaquine] for 7 days,
2. treatment [with Pyramax and high dose primaquine] for 14 days,
3. and a three-day treatment [3 days Pyramax and one day tafenoquine].

You had _______ treatment with us, right?

In addition to you, we had 960 participants in our study in Cambodia, but also in Ethiopia, Indonesia, and Pakistan.

(a) TQ: We found that with this treatment was the most effective. Very few patients who received it, had another malaria episode during follow up.
(b) PQ7: This treatment was a bit less effective than the three-day treatment [tafenoquine], but almost similar.

(c) PQ14: We found this treatment for 14 days was still effective just not as effective as the 7-day treatment and the three-day treatment. Approximately every 4th patient who received the treatment had another malaria episode within 6 months.

Do you have any questions about this so far?

Also, in terms of safety we had good results for all the treatment options. We found that no patients had serious events or reddish urine with the 7-day, or 3-day option and nausea and vomiting symptoms were mostly mild and limited. Tolerability of the 3-day option was better than the 7-day option.

Do you have any questions about this?

Ok, so I have now shared everything with you. We are finished, but if it is okay with you, I would like to give the phone to someone else so that you can answer a few questions about your satisfaction with our phone call and learning about the study results.

Response to refusal: No worries, thanks again for your participation in our study and for taking the time to talk with us today.

Response to agreement: Great, thank you. I will transfer you now.

File S3: Dissemination content guide for study team in Cambodia

EFFORT Patient Dissemination Discussion Guide

Hello _____, welcome. We are here today to discuss your experience with malaria treatment because you were a part of a study that provided treatment for your malaria.

1. Have you had malaria recently?
2. Can you describe your recent experiences with malaria treatment?
3. Do you remember being a part of study investigating malaria treatment?
4. What do you remember about this study?
5. What do you think about research or studies like these?

Now that we have completed the study you participated in, we are happy to be able to share the results with (all of) you, our participants. In addition to you, we had 960 participants in our study in Cambodia, but also in Ethiopia, Indonesia, and Pakistan.

You have all received a paper indicating the treatment you received and information about the study results. If you recall, you received one out of three types of treatment in this study. You may have received one of these 3 treatment options:

1. Treatment [with Pyramax and high dose primaquine] for 7 days,
2. Treatment [with Pyramax and high dose primaquine] for 14 days, and
3. A three-day treatment [3 days Pyramax and one day tafenoquine].

Do you remember what treatment options you received?

(d) TQ: In Cambodia, we found that with this treatment was the most effective. Very few patients who received it, had another malaria episode during follow up.
(e) PQ7: In Cambodia, we found that this treatment was a bit less effective than the three-day treatment [tafenoquine].
(f) PQ14: In Cambodia, we found this treatment for 14 days was still effective just not as effective as the 7-day treatment and the three-day treatment.

Do you have any questions about this so far?

Just to add to what I said before, the findings across not just Cambodia but the other three study countries also (Indonesia, Pakistan, and Ethiopia), was similar to Cambodia alone. But there was more of a difference in the effectiveness of the 7-day treatment vs the 14-day treatment. So, the 7-day and 3-day treatment were more clearly effective compared to the 14-day option.

Also, in terms of safety we had good results for all the treatment options. We found that no patients had a serious event or reddish urine with the 7-day, or 3-day option and nausea and vomiting symptoms were mostly mild and limited. Tolerability of the 3-day option was better than the 7-day option.

Do you have any questions about this?

Now, we will have a 5–10-minute break when you can have some of the refreshments and look at the paper with the study results. After that, we will start again, and you can share with us what you think of this process of sharing results today.

File S4: Trial handout provided to participants in Pakistan

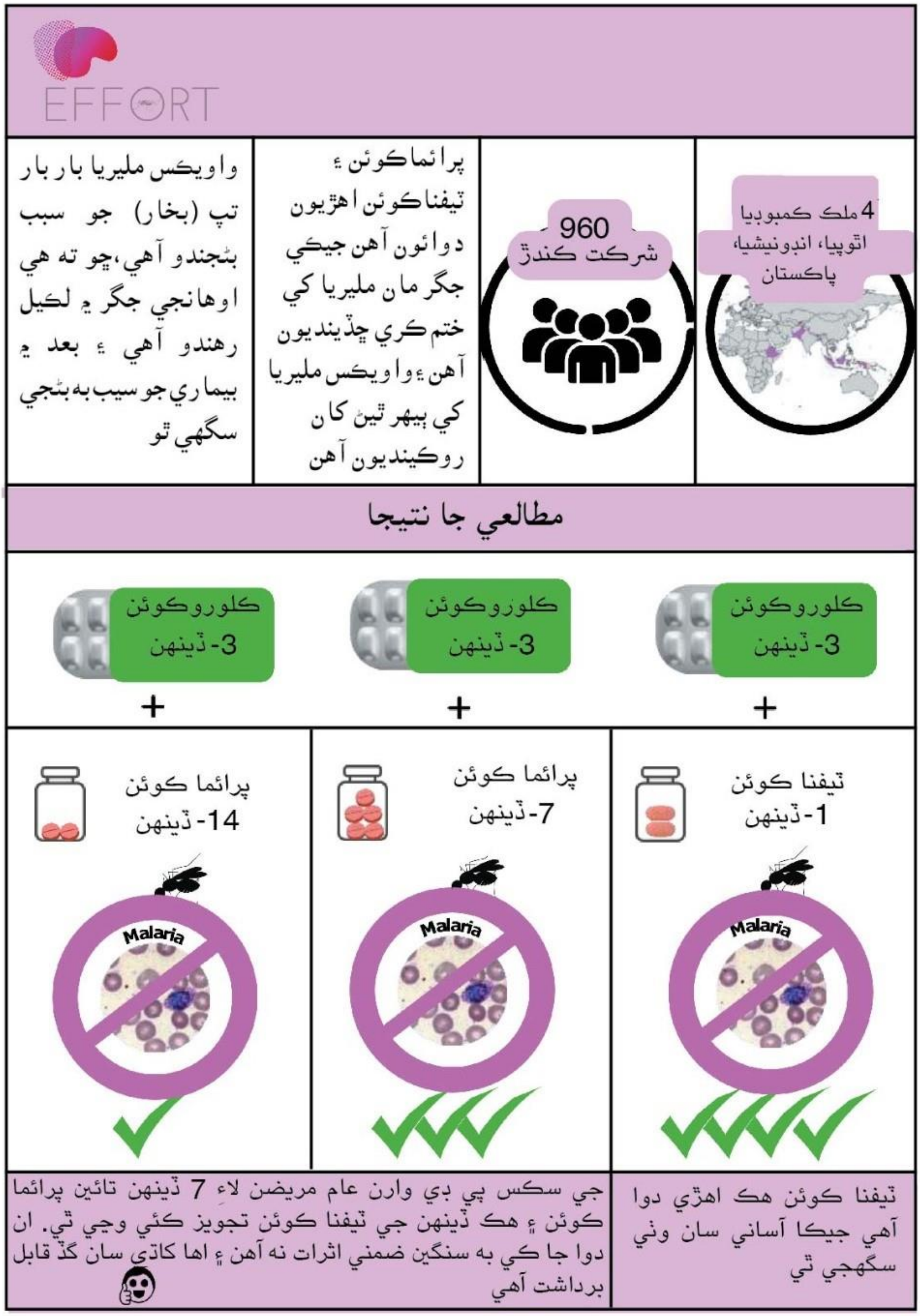

File S5: COREQ Guideline Checklist

COREQ (COnsolidated criteria for REporting Qualitative research) Checklist

Topic	Item No.	Guide Question/Description	Paper Reported on Page No.	Further information
**Domain 1: Research team and reflexivity**				
Personal Characteristics				
Interviewer/Facilitator	1	Which author/s conducted the interview or focus group?	Data collection section included for each study site country: p. 12-13	N/A
Credentials<br>Occupation<br>Gender<br>Experience & Training	2-5	• What were the researcher's credentials?<br>• What were the researcher's credentials? E.g. PhD, MD<br>• What was their occupation at the time of the study?<br>• Was the researcher male or female?<br>• What experience or training did the researcher have?	N/A	**Cambodia**: Data collectors are local study site staff (4 M & 2 F) with Bachelor level degrees that had previous experience in conducting focus group discussions and interviews and were supported by qualitative researchers with PhDs (one man and one women).<br><br>**Ethiopia**: Data collector (M) in Ethiopia has a MA in Social Anthropology and was supported by a PhD Candidate (F) with a Master of Science in Global Health with extensive experience in qualitative research.<br><br>**Pakistan**: Data collectors (F) are researchers and study staff at Aga Khan University with previous experience conducting focus group discussions. Lead facilitators have master's in public health.

Relationship to participants				
Relationship established	6	Was a relationship established prior to study commencement?	N/A	**Cambodia**: Respondents were familiar with data collectors given their previous interactions with study staff during the trial – however, this was not optimal for the evaluation, potentially introducing bias.<br><br>**Ethiopia**: Data collectors had previous experience with interviewing trial patients but did not have a relationship prior to the focus group discussions allowing for more open discussion about the dissemination experienced.<br><br>**Pakistan**: Data collectors had previous experience with interviewing trial patients but did not have a relationship prior to the focus group discussions besides making contact in person to invite participants to the focus group discussions.
Participant knowledge of the interview<br><br>Interviewer characteristics	7-8	What did the participants know about the researcher? e.g. personal goals, reasons for doing the research<br><br>What characteristics were reported about the interviewer/facilitator? e.g. Bias, assumptions, reasons and interests in the research topic	N/A	In all three countries, participants were aware that researchers wanted constructive feedback about the dissemination activities in which they had participated to make improvements in the future. The intentions of researchers were made clear at the start of the focus group discussions.
**Domain 2: Study Design**				
Theoretical Framework				

Methodological orientation and theory	9	What methodological orientation was stated to underpin the study? e.g. grounded theory, discourse analysis, ethnography, phenomenology, content analysis	Data analysis section of methods, description of analysis type and use/development of framework: p. 14 and Table S4	N/A
Participant selection				
Sampling	10	How were participants selected? e.g. purposive, convenience, consecutive, snowball	Study context: p. 6-12<br>Data collection section included for each study site country: p. 12-13	
Method of approach	11	How were participants approached? e.g. face-to-face, telephone, mail, email	Data collection section included for each study site country: p. 12-13	
Sample size	12	How many participants were in the study?	Data collection section included for each study site country: p. 12-13	
Non-participation	13	How many people refused to participate or dropped out? Reasons?	Data collection section included for each study site country: p. 12-13	
Setting				
Setting of data collection	14	Where was the data collected? e.g. home, clinic, workplace	Data collection section included for each study site country: p. 12-13	

Presence of non-participants	15	Was anyone else present besides the participants and researchers?	N/A	Only in Cambodia someone besides the researchers and participant was present – which was the baby/child of one of the facilitators and the children of some of the participants
Description of sample	16	What are the important characteristics of the sample? e.g. demographic data, date	Participant characteristic section of results: p. 15-16	N/A
Data collection				
Interview guide	17	Were questions, prompts, guides provided by the authors? Was it pilot tested?	Data collection section included for each study site country: p. 12-13	Given the limited sample size and parameters of the project, piloting was not possible. However, lessons learned from data collection in Cambodia were applied for Ethiopia and Pakistan.
Repeat interviews	18	Were repeat inter views carried out? If yes, how many?	N/A	N/A
Audio/visual recording	19	Did the research use audio or visual recording to collect the data?	Data collection section included for each study site country: p. 12-13	N/A
Field notes	20	Were field notes made during and/or after the inter view or focus group?	Data collection section included for each study site country: p. 12-13	N/A
Duration	21	What was the duration of the inter views or focus group?	N/A	Cambodia: 19 minutes - 1h 11m<br>Ethiopia:<br>Pakistan:
Data Saturation	22	Was data saturation discussed?	N/A	N/A
Transcripts returned	23	Were transcripts returned to participants for comment and/or correction?	N/A	This was not operationally possible

Domain 3: Analysis and findings				
Data Analysis				
Number of data coders	24	How many data coders coded the data?	Data analysis section of methods: p. 14	N/A
Description of the coding tree	25	Did authors provide a description of the coding tree?	Table S2	N/A
Derivation of themes	26	Were themes identified in advance or derived from the data?	Data analysis section of methods: p. 14	N/A
Software	27	What software, if applicable, was used to manage the data?	Data analysis section of methods: p. 14	N/A
Participant checking	28	Did participants provide feedback on the findings?	N/A	Member checking and validation was not operationally possible for this study
Reporting				
Quotations represented	29	Were participant quotations presented to illustrate the themes/findings? Was each quotation identified? e.g. participant number	Data collection section included for each study site country: p. 12-13, all sections of results include quotations: p. 15-30	An audit trail for data and quotations at the participant level was not possible due to a lack of differentiation between respondents in the transcript; however, an audit trail at the focus group discussion level was possible and included in the results.
Data and findings consistent	30	Was there consistency between the data presented and the findings?	All sections of results: p. 15-30	Yes.
Clarity of major themes	31	Were major themes clearly presented in the findings?	Results and discussion section: p. 15-36	Yes, themes were presented according to framework components.
Clarity of minor themes	32	Is there a description of diverse cases or discussion of minor themes?	Results and discussion section: p. 15-36	Yes.

Developed from: Tong A, Sainsbury P, Craig J. Consolidated criteria for reporting qualitative research (COREQ): a 32-item checklist for interviews and focus groups. International Journal for Quality in Health Care. 2007. Volume 19, Number 6: pp. 349 – 357

File S6: Phone dissemination satisfaction survey for participants in Cambodia

Satisfaction survey

Hello, I am _______, thank you for taking the time to answer a few short questions about your experience with this phone call. I will read you the questions and give you a few options for you to choose from. Do you mind if we record your answers? The recording will be deleted as soon as we enter the data. So your answers to the following questions will be anonymous.

Answer option 1: Yes, you can record.

Answer option 2: No, please do not record.

Ok, thank you. No problem. We can start.

1. How satisfied were you with how the study results were explained to you?
    a. Not satisfied
    b. Somewhat satisfied
    c. Satisfied
    d. Very satisfied

2. Do you have any suggestions of how we can improve?

3. Today, you were given two choices in how you'd like to receive the study results, in person or over the phone. Is there another way you would have preferred to receive the study results?
    a. No
    b. Yes

4. If, yes, what other way would you have preferred?

5. Was there any other information you would have liked to know about the study results?
    a. Yes
    b. No

6. If yes, what other information would you have liked to know about the study?

Okay, that was the last question. Thanks very much for your time. Goodbye.

File S7: Evaluation focus group discussion guide for study team in Cambodia

Evaluation Discussion Guide

1. How would you summarize the results that you heard today?

2. What do you think about how the study results were presented to you?

3. How satisfied were you with how the study results were explained to you?

4. If not satisfied with how the study results were explained to you, do you have any suggestions of how we can improve?

5. You were given two choices in how you'd like to receive the study results, in person or over the phone. Is there another way you would have preferred to receive the study results? And if so, what way would you have preferred?

6. Was there any other information you would have liked to know about the study results?

Okay, that was the last question. Thanks very much for your time. Goodbye.

File S8: Evaluation focus group discussion guide for study team in Ethiopia

Evaluation Discussion Guide

I. Welcome remarks

Introduce yourself and Muthoni (and make clear we are not part of the clinical trial team) Emphasize that this is an open free discussion, and that everything discussed here will remain confidential. There are no right or wrong answers. We just want to get your perspective on how the activity yesterday went so that we can know what was done well and what could be improved on for future studies.

II. Sign informed consent and patient information table

III. Confirm about recording

IV. Begin

1. Please introduce yourself

2. If you were to summarize the results to a friend or family member who was not present yesterday, what would you say?

    a. What messages or findings stood out to you the most?
    b. Were any findings surprising or new to you?

3. Was the information presented in a way that was easy to understand?

    a. Was any part of the presentation confusing or unclear?

4. What did you think about the format used (the PowerPoint presentation)?

    a. What could have been done differently to improve yesterday's activity?
    b. How would you prefer to receive such results in the future?

5. Did you feel there was enough time or opportunity to ask questions or share your thoughts?

6. What do you think should happen next with the results?

File S9: Evaluation focus group discussion guide for study team in Pakistan, English and Urdu

Evaluation Discussion Guide

Assalam o alaikum, my name is _________ and this is my team we welcome you all to the discussion today. We are here to discuss your experience with malaria treatment because you were a part of a study that provided treatment for your malaria. We will be recording the details during the session ONLY if you allow, ensuring your anonymity. There are no right and wrong answers to the questions we are just looking forward to having perspective of the community to have better treatment strategies in future.

6. Have you had malaria recently?
7. When you or a family member has a fever, what do you usually do first?
8. Can you describe your recent experiences with malaria treatment?
9. Where do you usually go for malaria treatment, and why do you prefer that place?
10. Have you ever used any home or traditional remedies for malaria before going to a health facility?
    a. If yes, what kind of remedies?
    b. Who recommends or provides them?
11. Do you usually complete the full course of malaria medication when prescribed? Why or why not?
12. What are some reasons people in your community might delay or avoid going to a health centre for malaria symptoms?
13. Have you or someone you know had a negative experience with malaria treatment at a health facility? If yes, can you describe what happened?
14. Who usually decides whether someone in your family should go for treatment when sick?
15. Do women and children in your household face any challenges in getting treatment when they are sick?
16. What do people in your village do to protect themselves from mosquitoes?
17. What do you think about research or studies like the one you participated in and heard the results about a few days ago?
18. What do you think about how the study results were presented to you?
    a. If not satisfied with how the study results were explained to you, do you have any suggestions of how we can improve?
    b. What did you think about the patient handout? Did you find it useful?
19. You were presented with the results in person and the dissemination event a few days ago. Is there another way you would have preferred to receive the study results?
    a. And if so, what way would you have preferred?
20. Was there any other information you would have liked to know about the study results?

21. If you were to explain the study results to your family or friends who did not attend the dissemination, what would you say?
22. Is there anything else you would like to share with us today?

Thank you for sharing your thoughts and experiences. Your feedback will help inform malaria control efforts in Thatta and other areas like it.

ملیریا علاج کے تجربات پر سوالنامہ

السلام علیکم، میرا نام __________ ہے اور یہ میری ٹیم ہے۔ ہم سب آپ کو آج کی گفتگو میں خوش آمدید کہتے ہیں۔ ہم یہاں آپ کے ملیریا کے علاج کے تجربے پر بات کرنے کے لیے آئے ہیں کیونکہ آپ اُس تحقیق کا حصہ تھے جس میں ملیریا کا علاج فراہم کیا گیا تھا۔ ہم اس نشست کے دوران صرف اُس صورت میں تفصیلات ریکارڈ کریں گے اگر آپ اجازت دیں گے، اور آپ کی شناخت صیغۂ راز میں رکھی جائے گی۔ ہمارے سوالات کے کوئی درست یا غلط جواب نہیں ہیں، ہم صرف کمیونٹی کے نقطہ نظر کو سمجھنا چاہتے ہیں تاکہ آئندہ بہتر علاج کی حکمتِ عملیاں بنائی جا سکیں۔

سوالات

1۔ کیا آپ کو حال ہی میں ملیریا ہوا ہے؟

2۔جب آپ یا آپ کے گھر میں کسی کو بخار ہوتا ہے تو آپ سب سے پہلے کیا کرتے ہیں؟

3۔کیا آپ اپنے حالیہ ملیریا کے علاج کے تجربے کے بارے میں بتا سکتے ہیں؟

4۔آپ عام طور پر ملیریا کے علاج کے لیے کہاں جاتے ہیں، اور آپ اس جگہ کو کیوں ترجیح دیتے ہیں؟

5۔کیا آپ نے کبھی کسی صحت کے مرکز میں جانے سے پہلے ملیریا کے لیے گھریلو یا روایتی علاج استعمال کیا ہے؟
.اگر ہاں، تو کس قسم کے علاج؟

انہیں کون تجویز کرتا ہے یا فراہم کرتا ہے؟

6۔جب آپ کو ملیریا کی دوا دی جاتی ہے تو کیا آپ عام طور پر پورا کورس مکمل کرتے ہیں؟ کیوں یا کیوں نہیں؟

آپ کی کمیونٹی میں لوگ کن وجوہات کی بنا پر ملیریا کی علامات کے باوجود صحت کے مرکز جانے میں تاخیر کرتے
اجتناب کرتے ہیں؟ 7۔ہیں یا

کیا آپ یا آپ کے کسی جاننے والے کو صحت کے مرکز میں ملیریا کے علاج کا منفی تجربہ ہوا ہے؟ اگر ہاں، تو کیا آپ بتا سکتے ہیں کہ کیا ہوا؟

9۔آپ کے خاندان میں عام طور پر یہ فیصلہ کون کرتا ہے کہ بیمار شخص کو علاج کے لیے جانا چاہیے یا نہیں؟

10۔کیا آپ کے گھر میں خواتین اور بچوں کو علاج کروانے میں کوئی مشکلات پیش آتی ہیں؟

11۔آپ کے گاؤں کے لوگ مچھروں سے بچاؤ کے لیے کیا کرتے ہیں؟

آپ اُس تحقیق یا مطالعے کے بارے میں کیا سوچتے ہیں جس میں آپ نے حصہ لیا اور جس کے نتائج آپ نے چند دن پہلےسنے؟

13۔ آپ کے خیال میں مطالعے کے نتائج آپ کو کس طرح پیش کیے گئے؟
اگر آپ مطمئن نہیں ہیں کہ نتائج کس طرح بیان کیے گئے، تو کیا آپ کے پاس کوئی تجاویز ہیں کہ ہم اس کو بہتر .
کیسے بنا سکتے ہیں؟

آپ نے مریضوں کے لیے جو معلوماتی پرچہ دیا گیا، اس کے بارے میں کیا سوچا؟ کیا یہ آپ کے لیے مفید تھا؟

آپ کو نتائج ذاتی طور پر اور ایک تقریب میں چند دن پہلے پیش کیے گئے تھے۔ کیا آپ کسی اور طریقے سے نتائج پسند کرتے؟ 14حاصل کرنا

اگر ہاں، تو کس طریقے سے؟

15۔کیا آپ کوئی اور معلومات جاننا چاہتے تھے جو نتائج میں شامل نہیں کی گئی تھی؟

اگر آپ کو اپنے گھر والوں یا دوستوں کو، جو اس تقریب میں شامل نہیں ہو سکے، مطالعے کے نتائج بتانے ہوں تو آپ کیا کہیں گے؟

17۔کیا آپ کچھ اور ہمارے ساتھ آج شیئر کرنا چاہتے ہیں؟

آپ کا شکریہ کہ آپ نے اپنے خیالات اور تجربات ہمارے ساتھ بانٹے۔ آپ کی رائے ملیریا کی روک تھام کی کوششوں میں، ٹھٹھہ اور اس جیسے دیگر علاقوں میں، مددگار ثابت ہوگی۔

Table S1: Joint analysis codebook

Code	Definition
Understanding of clinical trials	References demonstrating an understanding or lack thereof of clinical trials
> Expectations of clinical trials	References expressing expectations with regards to the clinical trials and being a part of them
> Individual vs public good	References to patients interests in personal results/participating in clinical trials for personal health reasons OR because they want to know the aggregate trial results and implications and are understanding of the public good benefit of clinical trial. This includes references in the context of dissemination content.
> Follow-up purpose	References speaking to patients understanding of the purpose of following up as part of clinical trials
Experience with clinical trials	References in which patients are describing their experience as part of the EFFORT clinical trial.
Trial treatment effectiveness/quality	References to the effectiveness or quality of treatment received in the trial e.g., references to the number of times participants have had malaria since the trial
Trial practice recommendations	References to patients explicit recommendations for trial practice or about dissemination of trial results but speaking to any improvement in clinical trial conduct
Description of dissemination	References describing clinical trial dissemination experienced
Dissemination activity	References in Cambodia data where dissemination is conducted
Impact of dissemination	Reference speaking to the impact of the dissemination on patient perceptions of clinical trials or understanding of results/the clinical trial
Knowledge transfer	References demonstrating patients understanding of clinical trial results
> Good knowledge transfer	References demonstrating patients clear understanding of clinical trial results
> Poor knowledge transfer	References demonstrating patients poor understanding of clinical trial results
Dissemination Perceptions	References expressing patients' opinion or emotions toward the result dissemination activities, including why certain patients may not want to come for dissemination or its evaluation
> Handout perceptions	References to perceptions regarding the patient dissemination handout
> Disseminations Recommendations	References identifying specific recommendations for the conduct of dissemination activities
> Dissemination Satisfaction	References expressing patients' satisfaction with the clinical trial result dissemination activities
Results meaning for policy	References speaking to the whether the treatments that were part of the study are now available for them, e.g., mentions of challenges for implementation of treatments in Ethiopia, or patients expressing the need for the treatment availability today in Cambodia.
Importance of personal recovery	References speaking to the importance of participants personal recovery above all

Role of patient dissemination	References speaking to patient’s role in the dissemination of clinical trial results
Public dissemination	References expressing the importance of clinical trial dissemination beyond participants to include the wide community, including recommendation for how this public dissemination should take place
Counselling thru Dissemination	References where dissemination is serving as an opportunity for general malaria counselling or an impact of the dissemination is improving patients’ malaria health literacy
Rumours	References to rumours/beliefs about clinical trials in the community, e.g., references to selling of blood, conspiracy theories, stigma caused, impact of dissemination on reducing/counteracting rumours/stigma
> 666	References to participants of EFFORT being a part the 666 conspiracy
> Selling of blood	References to perceptions of the clinical trial selling patients’ blood
Fear/Scepticism	References expressing or evoking patient/community fear or scepticism
Health literacy	References speaking to patients/community general health literacy
Interesting	References that I find interesting
Care seeking - PAK	References in Pakistan transcripts to general care seeking behaviour for malaria care
Societal norms - PAK	References in Pakistan transcripts to societal and family norms/dynamics relating to care seeking or treatment
Malaria treatment - PAK	References in Pakistan transcripts to general/routine malaria treatment provided
Malaria prevention - PAK	References in Pakistan transcripts to general malaria prevention practices used in communities
Traditional medicine - PAK	References in Pakistan transcripts to use of traditional medicine as treatment for malaria

Table S2: Development of components of the Research Dissemination Evaluation Framework

**Framework Component**	**Definition**	**Source**
Expectation for dissemination	What participants expect from the dissemination	Inductive code from this study: ‘individual vs public good’ speaking to expectations about receiving individual trial results<br><br>*Triangulated with*<br><br>Dissemination scoping review themes from Bagita et al. 2025
Motivation for dissemination attendance/desire	What motivates participants to join dissemination activities/desire for the dissemination	Inductive codes from this study: ‘perception of dissemination’ and ‘importance of personal recovering’ drawing on participants feelings towards receiving the results and wanting to participate in the dissemination to know how it affects their personal wellbeing and recovery – which was linked to participants expectation of receiving individual study results.
Affective attitude towards dissemination	Participants’ feelings towards the dissemination activities	Sekhon et al.’s 2017 acceptability framework<br><br>*Combined with*<br><br>Inductive code(s) from this study: ‘perception of dissemination’, ‘dissemination satisfaction,’ ‘handout perceptions’
Study result knowledge-transfer	Whether and what participants have understood of the study results communicated to them	Inductive code from this study: ‘knowledge transfer’
Affective attitude towards study results	Participants’ feelings towards results presented during the dissemination of activities	Sekhon et al.’s 2017 acceptability framework in terms of feelings and response towards the intervention<br><br>*Combined with*

		Concerns raised Bagita et al. 2025 about how participants receive the results
Impact of Dissemination	Effect or implications of dissemination for either participants or researchers	Inductive code from this study: ‘impact of dissemination’<br><br>*Combined with*<br>The reasons for dissemination theme from Bagita et al. 2025’s review
Subcomponent of Impact of Dissemination<br><br>Added Value	Benefits from dissemination beyond participants understanding the study result	Inductive code from this study: ‘impact of dissemination’<br><br>*Combined with*<br><br>Added value component of Cassidy-Seyoum et al. 2025’s revised feasibility and acceptability that drew on Sekhon et al.’s framework
Subcomponent of Added Value<br><br>Research understanding	Improvements in understanding of research more generally attributable to dissemination	Inductive code(s) from this study: ‘impact of dissemination,’ ‘understanding of clinical trials<br><br>*Combined with*<br><br>Component of Bagita et al. 2025’s review’s reasons for dissemination theme
Subcomponent of Added Value<br><br>Trust-building	Improvements in trust between researchers and communities or participants’ trust in the treatment/intervention of interest attributable to the dissemination	Inductive codes from this study: ‘impact of dissemination,’ ‘rumours’<br><br>*Triangulated with*<br><br>Component of Bagita et al. 2025’s review’s reasons for dissemination theme
Subcomponent of Added Value<br><br>Health literacy	Improvements in general health literacy, or specific to research, attributable to the dissemination	Inductive codes from this study: ‘impact of dissemination,’ ‘counselling thru dissemination’<br><br>*Triangulated with*

		Component of Bagita et al. 2025's review's reasons for dissemination theme
Subcomponent of Impact of Dissemination<br><br>Drawbacks	Negative outcomes of dissemination activities	Sekhon et al.'s acceptability framework components of opportunity cost and burden<br><br>*Combined with*<br><br>Concerns raised Bagita et al. 2025 about how participants receive the results
Subcomponents of Drawbacks<br><br>Opportunity cost	How much benefit, profit, or value must be given up to participate in the dissemination	Sekhon et al.'s acceptability framework component of opportunity cost
Subcomponents of Drawbacks<br><br>Emotional distress/stigma	Emotional distress or discomfort caused by the dissemination research results	Concerns raised Bagita et al. 2025 about how participants receive the results
Subcomponents of Drawbacks<br><br>Burden	How much effort is required to participate in the dissemination	Sekhon et al.'s acceptability framework component of burden
Dissemination recommendations	Participants recommendations for improving dissemination activities	Inductive codes from this study: 'dissemination recommendation,' 'dissemination perceptions'<br><br>*Combined with*<br><br>Bagita et al. 2025's review's themes of methods for dissemination, preferences for methods, and timing of dissemination

Subcomponents of Dissemination recommendations<br><br>Timing	When dissemination activities should take place	Inductive codes from this study: 'dissemination recommendation,' 'dissemination perceptions'<br><br>*Combined with*<br><br>Bagita et al. 2025's review's theme of timing of dissemination
Subcomponents of Dissemination recommendations<br><br>Mode	How study results should be disseminated	Inductive codes from this study: 'dissemination recommendation,' 'dissemination perceptions'<br><br>*Combined with*<br><br>Bagita et al. 2025's review's theme of methods for dissemination
Subcomponents of Dissemination recommendations<br><br>Implementer	Who should conduct the dissemination activities	Bagita et al. 2025's review's theme of preferences for dissemination

*Grey shading indicates framework components that have sub-components as illustrated in Figure 1 of the manuscript

Table S3: Details of study ethical approval

Institution, Country	Ethics number
Human Research Ethics Committee of the Menzies School of Health Research and the Northern Territory, Australia<br><br>John Mathews Building (Bldg 58)<br>Royal Darwin Hospital Campus, Rock<br>PO Box: 41096, Casuarina NT 0811, Australia<br>Website: www.menzies.edu.au	HREC-2020-3694
Oxford Tropical Research Ethics Committee (OxTREC), United Kingdom<br><br>University of Oxford<br>Research Services, University Offices<br>Willington Square, Oxford OX1 2JD<br>Tel: +44 (0) 1865 (2) 82106<br>E-mail: oxtrec@admin.ox.ac.uk<br>Website: www.admin.ox.ac.uk/rso/	Ref. 39-20
National Ethics Committee for Health Research, Cambodia<br><br>Lot #80, Samdach Penn Nouth Blvd (289), Sangkat Boeung Kok 2, Khan Tuol Kork,<br>Phnom Penh, Cambodia.<br>Tel: (855~12) 842 442, (855-012) 528 789, (855-012) 203 382	# 118
Arba Minch University Institutional Ethics Review Board, Ethiopia<br><br>PO Box 21, Arba Minch, Southern Region, Ethiopia<br>Tel: +251 46 8810414, 46 1817742, 46 8810070, 46 8810534<br>Email: irb.amu@yahoo.com	IRB/302/12
The National Research Ethics Review Committee (NRERC), Ethiopia<br><br>Addis Ababa, PO Box: 2490,<br>Tel: +251 114-674-353 Fax: +251 114-660-241<br>E-mail: most@ethionet.et<br>Website: www.most.gov.et	NRERC;<br>SRA/14.1/5311/20
The Ethiopian Food and Drug Authority, Addis Ababa, Ethiopia<br><br>Addis Ababa, PO Box: 5681,<br>Tel: 251-11-552 41 22/552 41 23 Fax: 251-11-552 13 92<br>E-mail: regulatory@fmaca.gov.et	EFDA;<br>02/25/33/05
Ethics Review Committee, Aga Khan University, Pakistan<br><br>Stadium Road, P.O. Box 3500 Karachi 74800, Pakistan.<br>Telephone: +92 21 3493 0051 Ext: 2447/4988.<br>Email: erc.pakistan@aku.edu	2022-7042-20769
National Institutes of Health, Health Research Institute, National Bioethics Committee (NBC)<br><br>Shahrah-e-Jamhuriat, Off Constitution Avenue, Sector G-5/2, Islamabad<br>Tel: 92-51-9224325, 9216793, Fax 9216774<br>www.nbcpakistan.org.pk,e-mail: nbcpakistan@nih.org.pk	No.4-87/NBC-772/22/ 1833
Drug Regulatory Authority of Pakistan<br><br>Pharmacy Services Division Prime Minister's National Health Complex, Park Road, Chak Shahzad, Islamabad, Pakistan<br>Telephone: +92 51 9107316, Email: ceo@dra.gov.pk	No. 03/10/2022-DD (PS)

Table S4: Specific suggestions for dissemination from participants

Suggestion Type	Description of suggestions	Rationale	Country
Communication strategies	Provision of dissemination materials electronically prior to the session	Preparation and getting the most out of the session	Ethiopia (*et-fgd5*)
	Making printed materials available during dissemination	Reference for the future and to improve participants' understanding	Ethiopia (*et-fgd1,3,5*)
	Smaller group sizes	Participants being more comfortable asking questions, having more time to ask questions, being able to read the slides better, and accommodate for late arrivals	Ethiopia (*et-fgd1,2,5,6*)
	Presentation slides in the local language	Enhance clarity and understanding for all participants	Ethiopia (*et-fgd1,3*)
	No use of technical language	Enhance clarity and understanding for all participants	Ethiopia (*et-fgd6*)
	More and clearer visuals in the presentation	Enhance clarity and understanding for all participants	Ethiopia (*et-fgd1,3*)
	Asking participants about their language needs prior to dissemination activities	Ensure that there are language accommodations and that everyone can understand the results	Ethiopia (*et-fgd2*)
	Organizing dissemination activities according to people's language needs, including sign language if needed	Ensure that everyone can understand the results	Ethiopia (*et-fgd2-4,6*)
	More time for presentations and question-and-answer sessions	Ensure clarity for all participants	Ethiopia (*et-fgd2,6*)
Mode of dissemination	Disseminate through in-person group meetings	The best way to have clear explanations and ask questions, allowing for livelier discussions	Cambodia (*cm-fgd2,3*)<br>Ethiopia (*et-fgd4,5*)
	Disseminate through participant home visits	Making it easier for patients	Cambodia (*Phone survey*)
Content of dissemination	Include malaria situation, whether novel treatments are available, and individual results as part of the dissemination content	Ensuring participants can best protect themselves from malaria and understand how study results relate to their health	Cambodia (*cm-fgd1, Phone survey*)<br>Ethiopia (*et-fgd4*)
Target of dissemination	Disseminate to the broader community beyond trial participants	Removing the stigma around the trial, wanting to share with others about the availability of free and effective treatment, improving malaria transmission, treatment, and prevention knowledge	Cambodia (*cm-fgd2,3*)<br>Ethiopia (*et-fgd1-6*)<br>Pakistan (*pk-fgd2,4*)
