# Supplementary material for: Dissemination of trial results to study participants in Cambodia, Ethiopia and Pakistan: a qualitative study
Source: BMJ Open. 2026 Aug 10;16(8):e119698. doi: 10.1136/bmjopen-2026-119698 (PMC13475131; doi:10.1136/bmjopen-2026-119698)
Supplement: Supplementary file 2 [file bmjopen-2026-119698.reviewer_comments.pdf]

PEER REVIEW HISTORY

BMJ Open publishes all reviews undertaken for accepted manuscripts. Reviewers are asked to complete a checklist review form and are provided with free text boxes to elaborate on their assessment. These free text comments are reproduced below.

ARTICLE DETAILS

Title (Provisional)

Dissemination of trial results to study participants in Cambodia, Ethiopia, and Pakistan: A Qualitative Study

Authors

Cassidy-Seyoum, Sarah A.; Adhikari, Bipin; Mwaura, Muthoni; Khan, Asia; Zaidi, Shan-E- Zehra; Azizullah, Zahida; Qurashi, Bushra; Gellebo, Kansite; Bamboro, Samuel Alemu; Ean, Mom; Tripura, Rupam; Vattanak, Hem; Vanda, Thy; Sovann, Kem; Simvieng, Ou; Chanpheakdey, Phal; Mnjala, Hellen; LEE, GRANT; Ghanchi, Najia; Cheah, Phaik Yeong; Price, Ric N; Beg, M. Asim; Degaga, Tamiru Shibiru; Dysoley, Lek; Thriemer, Kamala

---

VERSION 1 - REVIEW

**Reviewer**	**1**
**Name**	**van Delden, Johannes**
**Affiliation**	**Julius Center for Health Sciences and Primary Care**
**Date**	**07-Apr-2026**
**COI**	**None**

This is an interesting paper describing a qualitative study about disseminating trial results to participants in Cambodia, Ethiopia and Pakistan. The paper addresses a real problem: study results quite often do not reach participants, which goes against international guidelines such as DoH and CIOMS.

Having said that, I think the paper needs some serious rewriting.

1The paper fails to explain why this study was needed, given that there is already some evidence about the matter (line 98). Just adding data is not a rationale for any study (line 103).

2 Also, it is not clear to me how the study wants to address the issue of a lack of agreed practices (line 103). The results certainly do not speak to that.

3 In line with this: the stated objectives (line 121) are very general obscuring the fact that this in reality is a piggybag study (nothing wrong with that in itself) in which the dissemination activities of a specific study were studied. So what we get in the end are 75 (!!) pages of very detailed results about specific activities and how they were received. The research questions (or what I think could be read as such = the objectives) are not answered really. I think therefore, that there is a mismatch between the stated objectives and the results presented.

4 Also, the one result (is it a result?) that is more general (table 5, page 36) is presented in the discussion (sic) and comes out of nowhere. It is not at all clear how the content of the table relates to the findings. It feels like a list of good advice (which it generally is!) based on the expertise of the authors, but not as a result of the study.

5. I am sorry to say that nothing in the conclusion (page 38) stroke me as particularly new (nor was I particularly impressed with the strengths of this study presented on page 5). What have you really added to the literature after writing 75 pages of text?

6 I agree that dissemination do touch on PPIE but only if the process of dissemination involves at least some form of cocreation. Merely informing is not the same as PPIE (line 105). Please keep these notions apart, especially since in line 287 you admit that there was no PPIE in your own study. Why not, I wonder?

7 there are a lot of results (numbers of participants in a certain activity eg) in the methods, which I do understand to some degree (probably you want to prevent repetition), but I would still like you to reconsider whether

minor issues:

I understand that DoH 2013 was the current version at the time of collection of the data, but when you refer to the Doh in the introduction please you the 2024 version.

**Reviewer**	**2**
**Name**	**Martínez-López, María**
**Affiliation**	**University of Granada**
**Date**	**09-Apr-2026**
**COI**	**None**

I would like to thank the authors for this important and timely manuscript. It addresses a highly relevant yet still under-implemented ethical dimension of clinical research, namely the dissemination of study results to participants, and does so across multiple low- and middle-income settings. The study offers valuable empirical insights and practical reflections that could help inform future dissemination practices.

A key methodological issue is the substantial variation in dissemination formats and evaluation procedures across countries. Because the mode, timing, content, and evaluation process differed between sites, cross-country comparisons should be interpreted with caution. It would be helpful for the manuscript to foreground this limitation more explicitly.

The study may be affected by selection bias, as those who attended dissemination activities and agreed to participate in the evaluation were likely those most engaged with the trial or most positively disposed towards the research team. This is acknowledged in part, but I think its implications for the findings deserve further emphasis.

The possibility of social desirability bias should also be discussed more fully, particularly where dissemination and evaluation were conducted by the same or closely related study

teams. Participants may have felt reluctant to express dissatisfaction or criticism, especially in settings where trust and gratitude towards the team were prominent themes.

I would also encourage caution in the interpretation of knowledge transfer. Since no pre-dissemination baseline assessment was conducted, and in some settings the evaluation discussions themselves revisited or clarified the study results, it is difficult to determine how much understanding resulted from the dissemination activity itself. The manuscript may therefore wish to frame these findings more as perceived or observed indications of knowledge transfer rather than as firm evidence of effectiveness.

The proposed evaluation framework is potentially a valuable contribution. However, the manuscript would benefit from clarifying more explicitly how the framework was developed, how inductively it emerged from the data, and how it relates to pre-existing acceptability or dissemination frameworks. This would help readers better assess its originality and applicability.

Participant satisfaction is an important finding, but it may be useful for the authors to distinguish more clearly between satisfaction, ethical adequacy, and communicative effectiveness. A dissemination activity can be well received while still leaving important misunderstandings unresolved.

One of the most interesting findings is that some participants expected or hoped to receive individual results rather than only aggregate findings. I would encourage the authors to give this issue even greater prominence, as it raises important ethical and practical questions about what participants understand result dissemination to mean, and how aggregate findings relate to personal health expectations.

The recurring blurring between clinical care and research participation seems to me one of the most important findings of the study. It may be worth emphasising this more centrally, as it has implications not only for post-trial dissemination but also for informed consent, post-trial responsibilities, and trust in research.

The recommendations are useful and practical. However, I would suggest presenting them as context-informed recommendations rather than broadly generalisable best practices, given the specificity of the study settings and the heterogeneity of the dissemination approaches evaluated.

Overall, I found this manuscript thoughtful, relevant, and genuinely useful. It addresses an important ethical issue with care and practical sensitivity, and I very much enjoyed reading it.

**Reviewer**	**3**
**Name**	**Waligora, Marcin**
**Affiliation**	**Uniwersytet Jagiellonski w Krakowie Collegium Medicum, Department of Philosophy and Bioethics**
**Date**	**10-Apr-2026**
**COI**	**None**

A manuscript entitled “Dissemination of trial results to study participants in Cambodia, Ethiopia, and Pakistan: A Qualitative Study” aims to “evaluate and explore dissemination activities that were conducted as part of the multi-site clinical trial Plasmodium vivax treatment options, informing recommendations for best practices and providing a framework to guide future evaluations”. In my view, this aim is unfortunately not achieved.

Methods and crucial description of focus groups (FGs) are not provided, nor is there a description of the synthesis of FG results. A substantial part of the manuscript is unclear; the provided visual (Table 1) is inadequate, as it presents clinical trial site characteristics (not focus groups itself) instead of participants’ characteristics. My impression is that the results are presented in a rather random way, without appropriate data synthesis (no methods of thematic synthesis are provided). The references lack many significant works presenting the long-standing discussion about the need for dissemination of trial results. Overall, I consider this manuscript to require substantial revision and, unfortunately, recommend rejection of this version of the manuscript.

**Reviewer**	**4**
**Name**	**Clarke, Mike**
**Affiliation**	**Queen's University Belfast, Northern Ireland Methodology Hub**
**Date**	**16-Apr-2026**
**COI**	**None**

This is a generally well written, detailed and thorough report of an important piece of qualitative research. It contains a large amount of information and its length may be daunting for some readers, such that some shortening or the preparation of an executive summary which could be provided in the supplementary materials might help some readers and knowledge transfer for this study. The findings will be informative for a global audience and the materials that are included in the supplementary files are also likely to be useful resources and sources of ideas for others researchers.

I only have two suggestions for improvement:

Lines 150-152: It would be good to include more discussion of the heterogeneity in interest in receiving the trial results in the four countries ranging from 100% in Ethiopia to 0% in Indonesia. For instance, do the authors think that this is cultural or related to any differences in how patients were asked about their interest in receiving the results? This was also reported in reference 24 but I don't think it is fully discussed in that article and it is such a striking finding that readers of this paper are likely to want to know more without having to obtain and check that paper.

Lines 572-584: The statements about the availability of "individual results" are important and it would be good to have more information in this paper about what this would require for the EFFORT study and how feasible it would be to prepare and provide their own results to

each participant in the trial. It might also be worth expanding this paragraph to discuss whether an individual's results could be given to them after they have completed their participation in the trial or should wait until the full analysis and report of the trial is available.

VERSION 1 - AUTHOR RESPONSE

Reviewer 1:

This is an interesting paper describing a qualitative study about disseminating trial results to participants in Cambodia, Ethiopia and Pakistan. The paper addresses a real problem: study results quite often do not reach participants, which goes against international guidelines such as DoH and CIOMS.

Comment 1: The paper fails to explain why this study was needed, given that there is already some evidence about the matter (line 98). Just adding data is not a rationale for any study (line 103).

> **Response**: Based on existing evidence reviews, there is considerable data from high income countries regarding dissemination—most evidence coming from the United States and Europe. Very limited evidence and information is available regarding dissemination in resource-limited contexts, especially in the Asia Pacific. Our study is filling this gap and providing evidence from three resource-limited settings. The objective of the study has been updated to more clearly centre the low- and middle-income countries (LMICs)/resource-limited context. The amended objective in the abstract now reads (lines 31-33): "*The purpose of this study was to evaluate and explore dissemination activities that were conducted as part of a multi-site clinical trial of Plasmodium vivax treatment options**, informing future dissemination strategies in similar resource-limited settings***." In the introduction it now reads (lines 143-145): "*The objective of this qualitative study was to evaluate and explore the perceived value of dissemination activities **whilst informing future dissemination strategies in similar resource-limited settings***."

Comment 2: Also, it is not clear to me how the study wants to address the issue of a lack of agreed practices (line 103). The results certainly do not speak to that.

> **Response**: We have adjusted the objectives detailed in the manuscript to accurately reflect the study objectives and results. Please see our replies to your comment #1. Based on the evaluation conducted and feedback from participants, we have proposed recommendations that can be applied and adapted to similar resource-limited contexts. In line with this, we have moved Table 5 to the results section and added how we derived our recommendations in the methods section (lines 324-325): "*Based on participants' suggestions, their perceptions regarding dissemination, and feasibility considerations, context-informed dissemination recommendations were derived*." We have also updated the recommendations table (Table 4, formerly Table 5) (lines 656-658) to demonstrate how our recommendations come from the data presented by framework component. In this way, our objective of informing recommendations is achieved, while emphasizing the necessity of obtaining

participant preferences about dissemination throughout the study period.

Comment 3: In line with this: the stated objectives (line 121) are very general obscuring the fact that this in reality is a piggybag study (nothing wrong with that in itself) in which the dissemination activities of a specific study were studied. So what we get in the end are 75 (!!) pages of very detailed results about specific activities and how they were received. The research questions (or what I think could be read as such = the objectives) are not answered really. I think therefore, that there is a mismatch between the stated objectives and the results presented.

Response: We have adjusted the objectives detailed in the manuscript to accurately reflect the study objectives and results. As outlined in our reply to comment #2 the objective now clearly centres the specific context of low- and middle-income countries (LMICs)/resource-limited settings from which very limited data exists on dissemination, as compared to the United States and Europe. Additionally, we have removed the dissemination evaluation framework from the objective of the study, however, it remains a key output, providing additional value as highlighted by Reviewer 2. To further support this, we have added more detail about how the framework was developed in the supplementary material (Table S2).

Comment 4: Also, the one result (is it a result?) that is more general (table 5, page 36) is presented in the discussion (sic) and comes out of nowhere. It is not at all clear how the content of the table relates to the findings. It feels like a list of good advice (which it generally is!) based on the expertise of the authors, but not as a result of the study.

Response: We have moved Table 4 to the supplementary material (Table S4), moved Table 5 (now Table 4) to the results section, and added how we derived our recommendations in the methods section (lines 324-325): "*Based on participants' suggestions, their perceptions regarding dissemination, and feasibility considerations, context-informed dissemination recommendations were derived*." We have also updated Table 5 (now Table 4, lines 656-658) to demonstrate how our recommendations come from the data presented by framework component.

Comment 5: I am sorry to say that nothing in the conclusion (page 38) stroke me as particularly new (nor was I particularly impressed with the strengths of this study presented on page 5). What have you really added to the literature after writing 75 pages of text?

Response: In the context of LMICs/resource-limited setting, dissemination is not often conducted, and there is limited evidence regarding strategies to implement dissemination in these settings. Our study/findings offer guidance to other researchers planning on conducting study result dissemination in these contexts.

Comment 6: I agree that dissemination do touch on PPIE but only if the process of dissemination involves at least some form of cocreation. Merely informing is not the same as

PPIE (line 105). Please keep these notions apart, especially since in line 287 you admit that there was no PPIE in your own study. Why not, I wonder?

Response: We agree that PPI is not the same as consulting participants in the form of a survey regarding their preference. We have removed the additional sentence that reads "*Patients' preferences for dissemination, however, were incorporated into the design of the dissemination that was evaluated*," from the patient and public involvement section of the methods. While co-creation is slowly becoming a part of malaria clinical trial and implementation research, in the context of the clinical trial reported on, budgetary and practical constraint meant that co-creation after participant preferences were derived was not possible in this instance.

Comment 7: there are a lot of results (numbers of participants in a certain activity eg) in the methods, which I do understand to some degree (probably you want to prevent repetition), but I would still like you to reconsider whethe

Response: We agree that that there are what could be considered results in methods section. In the description of the dissemination in the methods, we present what may be seen as results; however, this is to provide necessary context to the evaluation. To address the reviewer's concerns, we have moved the description of the number of FGDs and participants into the participant characteristics section of the results. The paragraph now reads (lines 360-369): "*In Cambodia, the 25 respondents who participated in the dissemination were included in the evaluation. One FGD with five respondents (four patients and one family member) was conducted in Phnom Kravanh (cm-fgd1), while two FGDs with four and three respondents (cm-fgd2,3) were conducted in Siem Pang. An interview was also conducted with one participant in Phnom Kravanh (cm-i1). In Ethiopia, of the 200 participants who attended the meeting, 35 signed up, and 34 ultimately participated. Six FGDs were conducted (et-fgd1-6) with 5-7 respondents in each (total of 34 respondents). In Pakistan, of the 35 who were invited, 23 participated in four FGDs (pk-fgd1-4). There were two groups of six male respondents and two groups of female respondents, with six and five respondents, respectively.*"

Comment 8: I understand that DoH 2013 was the current version at the time of collection of the data, but when you refer to the Doh in the introduction please you the 2024 version.

Response: We have updated the language in the introduction to indicate that the obligation for dissemination was added to the Helsinki Declaration in 2013 while the citation is for the 2024 version (lines 100-102): "*Since 2013, the World Medical Association's Helsinki Declaration states that there is an obligation for medical research results to be published and disseminated, and specifically that research participants should have the option of being informed of study results [1].*"

<u>Reviewer 2:</u>

I would like to thank the authors for this important and timely manuscript. It addresses a highly relevant yet still under-implemented ethical dimension of clinical research, namely the dissemination of study results to participants, and does so across multiple low- and middle-income settings. The study offers valuable empirical insights and practical reflections that could help inform future dissemination practices.

Overall, I found this manuscript thoughtful, relevant, and genuinely useful. It addresses an important ethical issue with care and practical sensitivity, and I very much enjoyed reading it.

Comment 1: A key methodological issue is the substantial variation in dissemination formats and evaluation procedures across countries. Because the mode, timing, content, and evaluation process differed between sites, cross-country comparisons should be interpreted with caution. It would be helpful for the manuscript to foreground this limitation more explicitly.

> **Response**: This limitation is described in the discussion (lines 780-782): "*Second, different dissemination evaluation methods were used in the different study countries, making cross-country comparisons difficult*." It is also highlighted in the strengths and limitation section before the abstract (lines 74-77): "*A key strength of this study is that includes three different settings and contexts, providing richer data and opportunities for triangulation; however, different dissemination evaluation methods were used in the different settings, making cross-country comparisons difficult.*" However, we have made adjustments to highlight this difference in methods/methodologies and its impact more. In the data analysis subsection of the methods, we have added the following (lines 328-329): "*The different methodologies used across the different study sites were considered in the analysis of the results and cross-country comparison were made with caution*."

Comment 2: The study may be affected by selection bias, as those who attended dissemination activities and agreed to participate in the evaluation were likely those most engaged with the trial or most positively disposed towards the research team. This is acknowledged in part, but I think its implications for the findings deserve further emphasis.

> **Response**: We agree that this is a limitation of the study and have highlighted it in the limitations section of the discussion (lines 784-786): "*Fourth, in Ethiopia and Pakistan, participants who agreed to participate in the evaluation (FGDs) may have been those most interested in the study and understanding of it, influencing the results*." Given the concerns about the length of the manuscript raised by reviewer #1 and #4, additional emphasis on the selection bias has not been added.

Comment 3: The possibility of social desirability bias should also be discussed more fully, particularly where dissemination and evaluation were conducted by the same or closely related study teams. Participants may have felt reluctant to express dissatisfaction or criticism, especially in settings where trust and gratitude towards the team were prominent themes.

Response: Social desirability bias is concern and limitation especially in the Cambodian context. This was acknowledged in the limitations section of discussion (lines 782-784): "*Third, the dissemination and its evaluation took place at the same time by the same study team in Cambodia, and this could have introduced bias and limited participants' openness to share potentially critical feedback*." Furthermore, in the paragraph in the discussion discussing participant satisfaction, we added language around this bias a how it could have impacted reported satisfaction (lines 732-735): "*Participants were satisfied overall with both the dissemination process and the explanations provided. While social desirability bias could have impacted reported satisfaction, participants in our study did offer some suggestions to improve dissemination activities, showing some level of openness to report critically*."

Comment 4: I would also encourage caution in the interpretation of knowledge transfer. Since no pre-dissemination baseline assessment was conducted, and in some settings the evaluation discussions themselves revisited or clarified the study results, it is difficult to determine how much understanding resulted from the dissemination activity itself. The manuscript may therefore wish to frame these findings more as perceived or observed indications of knowledge transfer rather than as firm evidence of effectiveness.

Response: Indeed, this is an important limitation. It is highlighted as a study limitation in the last paragraph of the discussion section. We have now added to that section on lines 786 to 788 "*Fifth, a pre-dissemination baseline assessment of knowledge was not conducted,* ***limiting conclusions regarding the effectiveness of the dissemination in transferring knowledge***." The language in the abstract was also changed as suggested to the following (lines 42-44): "*Dissemination participants perceived having learned from the dissemination, demonstrating their knowledge of the trial and its results and attributing it to the dissemination*." Additionally, in the data analysis section of the methods (lines 330-333), we added: "*Additionally,* ***while knowledge transfer cannot be determined without a baseline assessment, knowledge regarding the study, its results, research, and malaria were explored and perceived knowledge transfer assessed. To assess participants' knowledge and perceived knowledge transfer****, the differences in content presented in the different study countries were considered*." Moreover, adjustments were made to the knowledge transfer section of the results: the section was re-titled (line 430) to "*Knowledge and perceived indications of knowledge transfer*." Lastly, language pertaining to knowledge transfer in the discussion was updated to the following (lines 664-665): "*Knowledge and indications of knowledge transfer varied by country, potentially reflecting differences in dissemination methodology*."

Comment 5: The proposed evaluation framework is potentially a valuable contribution. However, the manuscript would benefit from clarifying more explicitly how the framework was developed, how inductively it emerged from the data, and how it relates to pre-existing acceptability or dissemination frameworks. This would help readers better assess its originality and applicability.

Response: We have added a table in the supplement (Table S2) that details how the framework was developed and the origin of the framework components.

Comment 6: Participant satisfaction is an important finding, but it may be useful for the authors to distinguish more clearly between satisfaction, ethical adequacy, and communicative effectiveness. A dissemination activity can be well received while still leaving important misunderstandings unresolved.

Response: Thank you for flagging these important distinctions. Such distinctions are highlighted in the results (lines 430-484, 575-607) and discussion (lines 661 and 674) where participant satisfaction (affective attitude) is reported separately from communication effectiveness (knowledge and perceived knowledge transfer) and how the dissemination was not effectiveness in correcting all misconceptions. For example, in the discussion (lines 661-674): "*Respondents participating in our study were motivated to attend dissemination activities to receive study results with thepotential to inform their personal health. There was also some expectation of receiving individual results rather than aggregate trial results.* ***Knowledge and indications of knowledge transfer varied by country, potentially reflecting differences in dissemination methodology. There were some misunderstandings regarding the trial, its treatment, and results among participants in all countries—most notably a blurring of lines between the care provided during the trial and during routine care****. Dissemination impact included the improvement of participants' malaria health literacy and general understanding of research, as well as building trust in the trial and research generally.* ***Overall, participants were satisfied with the dissemination because they gained an understanding of the trial, its results, malaria prevention mechanisms; and because they could fully appreciate their contribution to the study and feel a part of something larger and highly valued.*** *Participants did, however, offer some recommendations regarding accessibility and content and emphasized the importance of in-person dissemination and public dissemination*." Given the length of manuscript, we have not added further emphasize on these distinctions.

Comment 7: One of the most interesting findings is that some participants expected or hoped to receive individual results rather than only aggregate findings. I would encourage the authors to give this issue even greater prominence, as it raises important ethical and practical questions about what participants understand result dissemination to mean, and how aggregate findings relate to personal health expectations.

Response: We agree that participants expectation of receiving individual results is an interesting finding. We discuss this expectation and its implications in the discussion (lines 683-698): "*Though including individual results was beyond the scope of our dissemination efforts, almost a quarter of participants surveyed as part of the clinical trial wanted to know their individual results [26], and this was an expectation that was reported among Ethiopian participants in the evaluation as well. Such a desire for individual results is in line with surveys conducted among participants in cancer, diabetes, and genetics research [44–46]. However, there may be feasibility and logistical challenges with providing participants with individual results post-trial*

completion. The need to include individual results as part of post-trial dissemination could be mitigated by clear and consistent communication regarding individual tests and their results during trial visits. Additionally, during post-trial dissemination, there may be opportunities to better relate aggregate results to participants' individual experiences, highlighting how individual patients fit within these aggregated results and thereby better addressing patients' motivation of knowing how the results inform their own health and wellbeing." Exploring this expectation and how it is linked to how participants perceive and understand dissemination was however beyond its scope. To acknowledge this, we have added a sentence to the limitation paragraph of the dissemination (lines 791-792) that reads, "*Seventh, further research is needed to explore participants expectation of receiving individual results and how that could be implemented*."

Comment 8: The recurring blurring between clinical care and research participation seems to me one of the most important findings of the study. It may be worth emphasising this more centrally, as it has implications not only for post-trial dissemination but also for informed consent, post-trial responsibilities, and trust in research.

Response: We agree that this blurred line is an important finding and hence we added more data regarding this into the results section (lines 477-484): "*For instance, in Pakistan and Ethiopia, questions arose as to why treatment was not provided to children (et-fgd6, pk-fgd1). Additionally, based on the quality of care provided by the clinical trial staff there was an expectation that free treatment would be provided for other diseases beyond the trial (et-fgd3,5,6pk-fgd2). The blurred line between routine and trial care is best described by respondents in Pakistan, stating: "P6: This system should continue, this centre. P1: Yes, when it was closed in between, we were very worried, so this system should continue" (pk-fgd1)*." This additional data now further supports the two paragraphs in the discussion on this important finding section (lines 709-731): "*A misconception that was not addressed by dissemination activities was the role of research or clinical trials relative to routine care. Some participants in Pakistan and Ethiopia described, expected, and recommended provision of health services beyond malaria care as part of the trial. As has been found in other contexts [48–50], given the quality of care received during the trial, participants wanted this care to continue and expand to include more of their community members. These blurred lines between trial and routine care are partially attributable to how research sites and health facilities are physically integrated and how well these research sites are embedded into communities and participants' health-seeking pathways, given their longstanding presence in the community conducting different research projects. Such an understanding likely reflects the need for clearer communication during the informed consent process, taking into consideration that this communication needs to be balanced against fuelling patients' fears of being experimented on.*

These blurred lines also bring into question the ethical responsibility clinical trial teams and researchers have in post-trial access and care [51]. There are conflicting views on the need or lack thereof to provide post-trial access, such as arranging access to investigational interventions [51]. However, proponents of post-trial access ground their support for it in equity and avoiding exploitation, especially in the context of LMICs, where participants might not have access to quality or required care outside of the clinical trial context [51-53]. For some, post-trial access should

go beyond arranging access to the clinical trial intervention to include responsible transitioning to needed healthcare [53–55]. Integrating post-trial care as a topic of discussion during the informed consent process would contribute to patients' understanding of the trial and help distinguish between the trial and routine services."

To emphasize it further we have added a reference to it in the abstract (lines 44-46): "*Dissemination activities may have failed to correct certain misconceptions, including the blurred distinction between clinical care and trial-related care*."

Comment 9: The recommendations are useful and practical. However, I would suggest presenting them as context-informed recommendations rather than broadly generalisable best practices, given the specificity of the study settings and the heterogeneity of the dissemination approaches evaluated.

Response: We have incorporated your feedback by updating the language throughout the manuscript: in the conclusion abstract (lines 48-49): "*Contributing the limited data on dissemination in resource-limited settings, our findings provide* ***context-informed recommendations*** *that can be applied to similar settings*" ; in the methods section (lines 324-325): "*Based on participants' dissemination suggestions, their perceptions regarding dissemination, and feasibility considerations,* ***context-informed dissemination recommendations*** *were derived*;" in the results section, (lines 654-655): "*Based on participant suggestions, perceptions of dissemination, and feasibility considerations,* ***context-specific recommendations*** *are reported in Table 4*;" and in the discussion section (lines 751-753): "*Study participants shared experiences and recommendations, which informed the general context-specific recommendations outlined in Table 5 that could be applied in similar resource-limited settings*."

<u>Reviewer 3:</u>

Overall, I consider this manuscript to require substantial revision and, unfortunately, recommend rejection of this version of the manuscript.

Comment 1: A manuscript entitled "Dissemination of trial results to study participants in Cambodia, Ethiopia, and Pakistan: A Qualitative Study" aims to "evaluate and explore dissemination activities that were conducted as part of the multi-site clinical trial Plasmodium vivax treatment options, informing recommendations for best practices and providing a framework to guide future evaluations". In my view, this aim is unfortunately not achieved.

Response: Please refer to the response to comments 2-4 from Reviewer 1.

Comment 2: Methods and crucial description of focus groups (FGs) are not provided, nor is there a description of the synthesis of FG results.

Response: We are not sure which crucial description you are referring to, however, in addition to the description of how the FGDs were conducted (lines 247-297), the discussion guides in the supplemental materials (File S6-9) and additional details we have in the supplemental material (File S5, Table S1); we have clarified the analysis/thematic analysis process and how it relates to creation of the research

dissemination evaluation framework (lines 312-325): "*All transcripts were reviewed (SCS) after a debrief with all study site teams. A thematic analysis was conducted with an inductive codebook (Table S1). Transcripts were imported into Dedoose and coded (SCS).* ***Memos were drafted based on themes emerging from a review of code excerpts (individual and combined) and reviewed by respective study site teams (BA, PC, EM, OS, MM, KG, AK, SZ, BQ). Themes included understanding of clinic trials, motivation and expectations for dissemination, knowledge transfer, impact of dissemination, fear and scepticism, and dissemination perceptions. These themes informed and were further developed by a derived theoretical framework to evaluate dissemination of research results (Fig 1, Table S2). In addition to study results (themes), the framework was derived from acceptability frameworks [40,41] and available literature regarding the implementation of dissemination (Table S2) [10]. Results are presented according to the derived framework components (Fig. 1), including participants' dissemination suggestions.***

Based on participants' dissemination suggestions, their perceptions regarding dissemination, and feasibility considerations, context-informed dissemination recommendations were derived."

Comment 3: A substantial part of the manuscript is unclear;

Response: We have reviewed the manuscript for clarity and addressed or incorporated feedback from our 4 reviewers—further adding clarity to the manuscript.

Comment 4: The provided visual (Table 1) is inadequate, as it presents clinical trial site characteristics (not focus groups itself) instead of participants' characteristics.

Response: While it is true that Table 1 provides site characteristics, which we believe is necessary to understanding the different country contexts, participant characteristics are detailed in Table 2 (lines 398-402) in the results section of the manuscript.

Comment 5: My impression is that the results are presented in a rather random way, without appropriate data synthesis (no methods of thematic synthesis are provided).

Response: The results are presented according to the evaluation framework developed as part of this study, illustrated in Figure 1. Further details on how this framework was developed have been added to the supplemental material (Table S2). Furthermore, we have added more clarity around the steps of the thematic analysis in the data analysis section (lines 312-325): "*All transcripts were reviewed (SCS) after a debrief with all study site teams. A thematic analysis was conducted with an inductive codebook (Table S1). Transcripts were imported into Dedoose and coded (SCS). Memos were drafted based on themes emerging from a review of code excerpts (individual and combined) and reviewed by respective study site teams (BA, PC, EM, OS, MM, KG, AK, SZ, BQ). Themes included understanding of clinic trials, motivation and expectations for dissemination, knowledge transfer, impact of dissemination, fear and*

scepticism, and dissemination perceptions. These themes informed and were further developed by a derived theoretical framework to evaluate dissemination of research results (Fig 1, Table S2). In addition to study results (themes), the framework was derived from acceptability frameworks [40,41] and available literature regarding the implementation of dissemination (Table S2) [10]. Results are presented according to the derived framework components (Fig. 1), including participants' dissemination suggestions.

Based on participants' dissemination suggestions, their perceptions regarding dissemination, and feasibility considerations, context-informed dissemination recommendations were derived."

More details regarding qualitative methodology are provided in the supplemental material (File S5), as is the inductive codebook (Table S1).

Comment 6: The references lack many significant works presenting the long-standing discussion about the need for dissemination of trial results.

Response: We have included references relevant to the context of community engagement and dissemination in the context of research in LMICs and the Helsinki Declaration Reference as well a large systematic review that summarizes current knowledge around dissemination (Bagita-Vangana, Plos med). In addition, we have also added two additional references, including Fernandez et al 2003 and Teichman et al. 2016.

Reviewer 4:

Comment 1: This is a generally well written, detailed and thorough report of an important piece of qualitative research. It contains a large amount of information and its length may be daunting for some readers, such that some shortening or the preparation of an executive summary which could be provided in the supplementary materials might help some readers and knowledge transfer for this study. The findings will be informative for a global audience and the materials that are included in the supplementary files are also likely to be useful resources and sources of ideas for others researchers.

Response: Thank you for your feedback. While we have shortened certain section of the manuscript e.g. the conclusion, we have added to balance shortening the manuscript with requests for additions to the manuscript from reviewers.

Comment 2: Lines 150-152: It would be good to include more discussion of the heterogeneity in interest in receiving the trial results in the four countries ranging from 100% in Ethiopia to 0% in Indonesia. For instance, do the authors think that this is cultural or related to any differences in how patients were asked about their interest in receiving the

results? This was also reported in reference 24 but I don't think it is fully discussed in that article and it is such a striking finding that readers of this paper are likely to want to know more without having to obtain and check that paper.

> **Response**: We agree that no participants wanting to receive results in Indonesia is a striking finding; however, this finding is from a previous study. Given the length of the current manuscript and its scope, we have added one sentence in the methods section (lines 174-178): "*Distribution was heterogeneous across study countries with 33.3% (58/174) expressing interest in Cambodia, 100% (334/334/) in Ethiopia, 97.7% (209/214) in Pakistan, and 0.0% (0/85) in Indonesia [26];* ***the lack of interest in Indonesia potentially explained by the costs of participating in dissemination and how it was communicated to participants***."

Comment 3: Lines 572-584: The statements about the availability of "individual results" are important and it would be good to have more information in this paper about what this would require for the EFFORT study and how feasible it would be to prepare and provide their own results to each participant in the trial. It might also be worth expanding this paragraph to discuss whether an individual's results could be given to them after they have completed their participation in the trial or should wait until the full analysis and report of the trial is available.

> **Response**: We agree that participants expectation of receiving individual results is a interesting finding. Please also see our reply to comment #7, reviewer #2.

References

1. Bagita-Vangana M, Unger HW, Thriemer K. Current global practice and implications for future research on disseminating health research results to study participants: A systematic review. PLOS Med. 2025 Aug 14;22(8):e1004569. doi:10.1371/journal.pmed.1004569

VERSION 2 - REVIEW

**Reviewer**	**1**
**Name**	**van Delden, Johannes**
**Affiliation**	**Julius Center for Health Sciences and Primary Care**
**Date**	**19-Jun-2026**
**COI**	

The content of the paper is now better aligned with the objectives. I appreciate that the suggestions for improvement have been moved to the appendix.

However, I still think the paper is way too long. This discussion is a chapter! Please shorten the paper considerably by stating the main learnings and not commenting on every detail

Reviewer 2

Name Martínez-López, María

Affiliation University of Granada

Date 30-Jun-2026

COI

I would like to thank the authors for the considerable effort they have made in revising this manuscript. I believe the revised version has improved substantially and that the vast majority of my previous comments have been thoughtfully addressed.

In particular, I appreciate the additional methodological transparency regarding the development of the dissemination evaluation framework. The inclusion of the coding framework and the detailed explanation of how the framework components were derived and integrated with existing conceptual models greatly strengthen the manuscript and make its contribution much clearer.

I also appreciate the greater emphasis placed on participants' expectations regarding individual versus aggregate results, which I consider one of the most interesting and ethically important findings of the study.

While I still believe that some aspects—such as the interpretation of knowledge transfer and the potential influence of selection and social desirability bias—could be discussed in slightly greater depth, these are relatively minor points that do not detract from the overall quality of the manuscript.

Overall, I found the revised manuscript to be significantly strengthened, and I would like to congratulate the authors on this valuable contribution to an important and often overlooked area of research ethics.

Reviewer 3

Name Waligora, Marcin

Affiliation Uniwersytet Jagiellonski w Krakowie Collegium Medicum, Department of Philosophy and Bioethics

Date 23-Jun-2026

COI

The manuscript has been substantially improved. My only suggestion is to expand the part about dissemination of clinical trials results. I suggest to consider including results of other research: 1)

Wieschowski S, Riedel N, Wollmann K, et al. Result dissemination from clinical trials conducted at German University medical centers was delayed and incomplete. J Clin

Epidemiol 2019;115:37–45. 10.1016/j.jclinepi.2019.06.002; 2) Strzebonska K, Wasylewski MT, Zaborowska L, Riedel N, Wieschowski S, Strech D, Waligora M. Results dissemination of registered clinical trials across Polish academic institutions: a cross-sectional analysis. BMJ Open. 2020 Jan 22;10(1):e034666.; 3) Chen R, Desai NR, Ross JS, et al. Publication and reporting of clinical trial results: cross sectional analysis across academic medical centers. BMJ 2016;352:i637 10.1136/bmj.i637.

**Reviewer**	**4**
**Name**	**Clarke, Mike**
**Affiliation**	**Queen's University Belfast, Northern Ireland Methodology Hub**
**Date**	**09-Jul-2026**
**COI**	

Thank you for addressing my comments, and those of the other three peer reviewers. I am happy with the revised manuscript.

However, I remain concerned that its length will make knowledge transfer difficult. I recommend that you prepare an executive summary (perhaps 2-3 pages long) to provide your key messages in a much shorter form.

VERSION 2 - AUTHOR RESPONSE

Reviewer 1:

The content of the paper is now better aligned with the objectives. I appreciate that the suggestions for improvement have been moved to the appendix.

Comment 1: However, I still think the paper is way too long. This discussion is a chapter! Please shorten the paper considerably by stating the main learnings and not commenting on every detail

> **Response**: Thank you for taking the time to re-review our manuscript. We understand your concern about the length of the manuscript; however, we aimed to balance feedback from reviewer #2 and #3 asking for additional details to be added. That said, we have shortened the manuscript in certain areas: in the methods section we have shorted the description of the dissemination in each country, instead summary it in a new Table 2 (lines 174-176); we have also removed some details in the results sections (line 238 - page 19, lines 536, 538, 544 - page 23, line 577 – page 24, line 601 – page 25).

Reviewer 2:

Comment 1: I would like to thank the authors for the considerable effort they have made in revising this manuscript. I believe the revised version has improved substantially and that the vast majority of my previous comments have been thoughtfully addressed.

In particular, I appreciate the additional methodological transparency regarding the development of the dissemination evaluation framework. The inclusion of the coding framework and the detailed explanation of how the framework components were derived and integrated with existing conceptual models greatly strengthen the manuscript and make its contribution much clearer.

I also appreciate the greater emphasis placed on participants' expectations regarding individual versus aggregate results, which I consider one of the most interesting and ethically important findings of the study.

While I still believe that some aspects—such as the interpretation of knowledge transfer and the potential influence of selection and social desirability bias—could be discussed in slightly greater depth, these are relatively minor points that do not detract from the overall quality of the manuscript.

Overall, I found the revised manuscript to be significantly strengthened, and I would like to congratulate the authors on this valuable contribution to an important and often overlooked area of research ethics.

> **Response**: Thank you for taking the time to re-review our manuscript. We understand your request for the interpretation of knowledge transfer and the potential influence of selection and social desirability bias to be discussed more in depth. Given the length of the manuscript and comments regarding length from other reviewers, we have strengthened the language in the limitation paragraph of the discussion: *Third, the dissemination and its evaluation took place at the same time by the same study team in Cambodia, and this* ***likely*** *introduced bias and limited participants' openness to share potentially critical feedback* ***and favouring expression of satisfaction with the*** **dissemination"** (Lines 798-801); "*Fourth, in Ethiopia and Pakistan, participants who agreed to participate in the evaluation (FGDs) may have been those most interested in the study and understanding of it, influencing the results* ***regarding level of knowledge about the study and its results***" (Lines 801-803).

Reviewer 3:

Comment 1: The manuscript has been substantially improved. My only suggestion is to expand the part about dissemination of clinical trials results. I suggest to consider including results of other research: 1)

Wieschowski S, Riedel N, Wollmann K, et al. Result dissemination from clinical trials conducted at German University medical centers was delayed and incomplete. J Clin Epidemiol 2019;115:37–45. 10.1016/j.jclinepi.2019.06.002; 2) Strzebonska K, Wasylewski MT, Zaborowska L, Riedel N, Wieschowski S, Strech D, Waligora M. Results dissemination of registered clinical trials across Polish academic institutions: a cross-sectional analysis. BMJ Open. 2020 Jan 22;10(1):e034666.; 3) Chen R, Desai NR, Ross JS, et al. Publication and reporting of clinical trial results: cross sectional analysis across academic medical centers. BMJ 2016;352:i637 10.1136/bmj.i637.

Response: Thank you for taking the time to re-review our manuscript.We have updated the manuscript to include two of the suggested references. We have added it on the following lines 86-89: "*Dissemination of study results to participants as part of community engagement is increasingly recognised as an ethical and moral imperative in clinical research [1,10–12], and included in ethics guidelines [13]; however, in practice it is rarely implemented globally [10,11,14–16],* ***including in the context of high-income countries*** *[17,18].*"

Reviewer 4:

Comment 1: Thank you for addressing my comments, and those of the other three peer reviewers. I am happy with the revised manuscript.

However, I remain concerned that its length will make knowledge transfer difficult. I recommend that you prepare an executive summary (perhaps 2-3 pages long) to provide your key messages in a much shorter form.

Response: Thank you for taking the time to re-review our manuscript.. We understand your concern about the length of the manuscript; however, we adding an executive summary is not in line with the formatting guidelines of the journal. Should it be requested from the editorial team, we would be happy to add it. That said, we have shortened the manuscript in certain areas: in the methods section we have shorted the description of the dissemination in each country, instead summary it in a new Table 2 (lines 174-176); we have also removed some details in the results sections (line 238 - page 19, lines 536, 538, 544 - page 23, line 577 – page 24, line 601 – page 25).
